# Engineering exosomes for Alzheimer's disease: Multi‐target therapeutic strategies from pathogenesis to clinical translation

**DOI:** 10.1002/ctm2.70548

**Published:** 2025-12-15

**Authors:** Yuehan Zhang, Zhenyang Li, Haien Guan, Zhenhua Qiu, Chunlin Zou

**Affiliations:** ^1^ Center of Stem Cell and Regenerative Medicine The People's Hospital of Gaozhou Guangdong China; ^2^ Key Laboratory of Longevity and Aging‐Related Disease of Chinese Ministry of Education Institute of Neuroscience and Guangxi Key Laboratory of Brain Science, Center for Translational Medicine School of Basic Medical Sciences Guangxi Medical University Nanning China; ^3^ Key Laboratory of Human Development and Disease Research (Guangxi Medical University) Education Department of Guangxi Zhuang Autonomous Region Nanning China

**Keywords:** Alzheimer's disease, blood‒brain barrier, clinical translation, engineered exosomes, multi‐target therapy, safety assessment, targeted delivery

## Abstract

The complex pathogenesis of Alzheimer's disease (AD), combined with the presence of the blood‒brain barrier (BBB), severely limits the effectiveness of conventional therapeutic approaches. Engineered exosomes—nanoscale extracellular vesicles of natural origin—have emerged as a promising platform for innovative AD therapy due to their excellent biocompatibility, low immunogenicity and intrinsic ability to cross the BBB. This review provides a systematic overview of the synthetic and structural biological characteristics of exosomes, with a focus on their functionalisation through physical, chemical and genetic modifications. These approaches enable the targeted loading of therapeutic cargo and the conjugation of brain‐targeting peptides, thereby facilitating precise delivery to specific brain regions and offering a multi‐target therapeutic strategy for AD. We further examine the potential of engineered exosomes in modulating core AD pathological pathways, including amyloid‐beta deposition, tau hyperphosphorylation, neuroinflammation and synaptic dysfunction, and highlight their utility as an integrated delivery system for the co‐delivery of multiple therapeutic agents to achieve synergistic therapeutic effects. Finally, key challenges in clinical translation are addressed, such as scalable production, standardised drug loading protocols and comprehensive assessment of safety and immunogenicity. Unlike previous reviews that primarily focus on general engineering techniques, this article emphasises a rational design strategy tailored for multi‐target synergistic therapy and presents a comprehensive roadmap from basic research to clinical application, thereby providing both theoretical insights and practical guidance for the development of next‐generation AD treatments.

**Key points:**

- A multidimensional approach combining physical, chemical, and genetic modifications equips exosomes with brain‐targeted peptides, enhancing their capability for precise brain delivery in Alzheimer's disease (AD)
- Engineered exosomes are designed to cross the blood‐brain barrier and provide stimuli‐responsive release of therapeutic agents, enabling simultaneous clearance of amyloid‐beta plaques and neurofibrillary tangles, and inhibition of neuroinflammation.
- The transition from preclinical success to early‐phase human trials is underway, with intranasal administration emerging as a promising, non‐invasive method for brain drug delivery.
- A well‐defined plan for clinical translation includes scalable Good Manufacturing Practice (GMP) production, rigorous safety assessments, and biomarker‐guided clinical trial design to facilitate clinical application.

## Graphical abstract

Exosomes, modified through multidimensional approaches including physical, chemical and genetic means, and equipped with brain‐targeted peptides and therapeutic agents, are capable of crossing the blood‒brain barrier for precise delivery. They can simultaneously remove amyloid‐beta plaques and neurofibrillary tangle, inhibit neuroinflammation, and pave the way for a novel, multi‐dimensional therapeutic paradigm in neurodegenerative diseases.

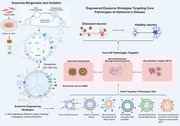

## 1 INTRODUCTION

Alzheimer's disease (AD), a devastating neurodegenerative disorder, is imposing a growing societal burden as its global prevalence continues to increase.^1^, ^2^ Current treatment options, including recently developed monoclonal antibodies targeting amyloid‐beta (Aβ), provide only limited symptomatic relief or modest disease‐modifying effects, and are often hampered by significant side effects and an inability to halt disease progression.^3^, ^4^ The multifaceted pathology of AD—driven by interconnected mechanisms such as Aβ plaque accumulation,^5^ tau hyperphosphorylation,^6^ chronic neuroinflammation,^7^ mitochondrial dysfunction,^8^ synaptic dysfunction^9^ and neuronal loss—underscores the urgent need for novel therapeutic approaches capable of simultaneously addressing multiple pathological pathways.^10^

A major obstacle in AD therapy is the blood‒brain barrier (BBB), which effectively excludes over 98% of small‐molecule drugs and nearly all large‐molecule therapeutics from entering the central nervous system (CNS).^11^, ^12^ Thus, the development of a delivery system that can safely and efficiently cross the BBB and precisely transport therapeutics to affected brain regions is critical for advancing AD treatment.

In this context, exosomes—endogenous nanoscale vesicles naturally released by cells—have attracted considerable interest due to their favourable biological properties.^13^, ^14^, ^15^ These include high biocompatibility,^16^ low immunogenicity^17^ and a natural ability to traverse the BBB.^18^ Importantly, exosomes can be engineered via genetic or chemical modification to display brain‐targeting peptides (such as the rabies virus glycoprotein‐derived RVG peptide^19^) on their surface and to encapsulate a diverse range of therapeutic cargo, including β‐site amyloid precursor protein (APP)‐cleaving enzyme 1 (BACE1)‐targeting siRNA, anti‐inflammatory microRNAs and neurotrophic factors such as brain‐derived neurotrophic factor (BDNF).^20^, ^21^ This adaptability positions engineered exosomes not merely as natural biological carriers, but as versatile and programmable platforms for targeted therapy.

Existing studies have primarily focused on the fundamental biology of exosomes, isolation techniques or general engineering approaches.^22^, ^23^ Although some literature has also explored the multi‐target potential of exosomes in AD,^24^, ^25^ a systematic and actionable framework for their rational design remains notably lacking—one that spans the entire pipeline from biogenesis and structural function to physical, chemical and genetic modifications, as well as scalable production, quality control and regulatory compliance. At present, there is an urgent need for a comprehensive review that integrates engineered exosomes for multi‐target synergistic therapy with the entire clinical translation workflow.

The novelty of this review lies in two key aspects: (i) establishing design principles for multi‐target synergistic therapy based on pathology—systematically elaborating functional strategies for exosomes in drug loading, targeting and spatiotemporal release by integrating key AD pathways such as Aβ, tau, neuroinflammation and mitochondrial dysfunction; and (ii) presenting a complete clinical translation roadmap—including the latest advances in Good Manufacturing Practice (GMP)‐compliant scalable production, quality standards, regulatory requirements and safety/immunogenicity assessments—thereby providing an actionable blueprint for the industrial development of next‐generation AD treatments.

Through this structured analysis, this review aims to offer researchers a holistic perspective that guides the reader from basic biology, through functional engineering and multi‐target efficacy, and finally to clinical translation, thereby helping to advance engineered exosomes towards next‐generation therapies for multi‐pathway intervention in AD.

## 2 PATHOGENIC MECHANISMS OF AD AND CURRENT STRATEGIES FOR SINGLE‐TARGET PHARMACOTHERAPY

### 2.1 AD pathogenic mechanisms

AD is no longer regarded as a condition with a singular cause, but rather as a complex network of interrelated pathological processes. As summarised in Table 1, this network is driven by several core mechanisms—primarily the Aβ cascade and tau pathology—which are critically modulated and exacerbated by neuroinflammation,^7^ mitochondrial dysfunction^8^ and metabolic dysregulation.^26^ AD pathogenesis often begins with a self‐reinforcing cycle involving the accumulation of Aβ oligomers and the hyperphosphorylation of tau protein. Neuroinflammation acts as a powerful disease amplifier: activated microglia, initially recruited to clear Aβ, may become dysfunctional and release pro‐inflammatory cytokines that further damage neurons and impede Aβ clearance.^27^ Meanwhile, mitochondrial dysfunction disrupts cellular energy production, elevates oxidative stress, and can directly promote both Aβ production and tau pathology. This establishes a vicious cycle, as Aβ and pathological tau further impair mitochondrial function, accelerating neuronal loss.^28^ The intricate interplay among these pathways explains why therapies targeting a single mechanism have largely failed. The robustness of the AD pathogenic network, sustained by multiple compensatory and reinforcing pathways, means that inhibiting one node often allows the disease to advance through others.^29^ Therefore, a comprehensive understanding of AD pathogenesis that integrates the interplay among distinct mechanisms is crucial for elucidating its progressive nature.

**TABLE 1:** Pathological mechanisms of Alzheimer's disease (AD) and therapeutic targets.

Pathological mechanism	Pathological process	Function	Key molecules	Therapeutic targets	Research status
Amyloid cascade hypothesis	Aβ overproduction, oligomerisation, aggregation into plaques, and impaired clearance^5^, ^30^, ^31^	Synaptic dysfunction, cognitive decline	APP, Aβ1‐40/42, γ‐secretase, RAGE receptor, ApoE4 isoform, BACE1 (β‐secretase), LRP1	Aβ (clearance: Aducanumab)^32^ γ‐Secretase (inhibitor: Semagacestat)^33^ APOE4 (gene editing: CRISPR/Cas9)^34^	Aducanumab: FDA‐approved (accelerated pathway, post‐marketing trials ongoing) Semagacestat: discontinued (phase III, safety issues) APOE4 CRISPR: preclinical (in vitro/animal models)
Tau hyperphosphorylation	Hyperphosphorylation of Tau protein leads to its dissociation from microtubules, oligomerisation and formation of NFTs^6^, ^35^	Neuronal degeneration, axonal transport failure	Tau protein, GSK‐3β, CDK5, PP2A	Pathological Tau species (monoclonal antibody: Gosuranemab)^36^ GSK‐3β (inhibitor: lithium)^37^ CDK5 (inhibitor: Roscovitine)^38^	Gosuranemab: negative results terminated development Lithium: phase II/III trials completed (no clear efficacy in AD, limited by toxicity) Roscovitine: preclinical (neuroprotective effects in models)
Neuroinflammation	Microglial activation and pro‐inflammatory cytokine release^7^, ^39^, ^40^	Synaptic pruning defects, oxidative stress, accelerated neuronal death	Microglia, astrocytes, TNF‐α, IL‐1β, TREM2, NLRP3 inflammasome, C1q, C3, CX3CL1	TREM2 (agonist: AL002)^41^ NLRP3 inflammasome (inhibitor: MCC950)^42^ IL‐1β (antibody: Canakinumab)^43^	AL002: phase I/II (NCT04592874) MCC950: preclinical (efficacy in AD models) Canakinumab: repurposed (approved for autoinflammatory diseases; AD trials ongoing)
Mitochondrial dysfunction	Dysregulation of mitochondrial dynamics (fission/fusion) and impairment of electron transport chain function^8^, ^44^	Energy failure, ROS accumulation, neuronal apoptosis	Aβ peptides, Tau protein, Fis1, Mfn2, DRP1, PGC‐1α	Mfn2 (activator: Leupeptin)^45^ Fis1 (inhibitor: P110)^46^ ROS (scavenger: CoQ10)^47^	Leupeptin: preclinical (animal models) P110: preclinical (mitochondrial fission inhibition) CoQ10: phase III trials completed (no significant cognitive benefit in AD)
Metabolic dysregulation	Brain insulin resistance, lipid peroxidation^26^, ^48^, ^49^	Neuronal energy deficiency, impaired synaptic signalling	Insulin, cholesterol, BCAAs, NMDAR, GLUT1, ApoJ, mTOR	Insulin receptor (intranasal insulin)^50^ NMDAR (modulator: memantine)^51^ Cholesterol metabolism (statins)^52^	Regular insulin: phase II (NCT00438568) Memantine: FDA approved (moderate‒severe AD) Statins: observational studies (conflicting evidence for AD prevention)
Genetic and epigenetic factors	Gene mutation‐driven pathology, epigenetic dysregulation^53^, ^54^	Early‐/late‐onset AD risk, impaired neuroprotective pathways	PSEN1/2, APOE4, BIN1, DNA methylation, histone acetylation, SORL1, miRNA	APOE4 (gene therapy: AAV‐APOE2)^55^ HDAC (inhibitor: Vorinostat)^56^	AAV‐APOE2: phase I (NCT03634007) Vorinostat: phase I (NCT03056495)
Gut microbiota‒brain axis	Gut dysbiosis‐induced systemic inflammation^57^	BBB disruption, reduced Aβ clearance, cognitive decline	Firmicutes, LPS, SCFAs, TMAO, bacteroidetes, Zonulin	LPS (inhibitor: Rifaximin)^58^ SCFAs (probiotics: VSL#3)^59^ TMAO (blocker: 3,3‐dimethyl‐1‐butanol)^60^	Rifaximin: phase II (NCT03856359) VSL#3: phase II (NCT00852124) 3,3‐Dimethyl‐1‐butanol: preclinical (TMAO reduction in models)

Abbreviations: Aβ, amyloid‐beta; APP, amyloid precursor protein; BACE1, β‐site APP‐cleaving enzyme 1; BBB, blood‒brain barrier; BCAAs, branched‐chain amino acids; BIN1, bridging integrator 1; CDK5, cyclin‐dependent kinase 5; CRISPR/Cas9, clustered regularly interspaced short palindromic repeats/CRISPR‐associated protein 9; CX3CL1, C‐X3‐C motif chemokine ligand 1; DRP1, dynamin‐related protein 1; Fis1, mitochondrial fission 1 protein; GLUT1, glucose transporter 1; GSK‐3β, glycogen synthase kinase‐3β; HDAC, histone deacetylase; LPS, lipopolysaccharide; LRP1, low‐density lipoprotein receptor‐related protein 1; mTOR, mammalian target of rapamycin; NFTs, neurofibrillary tangles; NLRP3, NACHT, LRR and PYD domains‐containing protein 3; NMDAR, N‐methyl‐D‐aspartate receptor; PP2A, protein phosphatase 2A; PSEN1/PSEN2, presenilin 1/presenilin 2; RAGE, receptor for advanced glycation endproducts; ROS, reactive oxygen species; SCFAs, short‐chain fatty acids; SORL1, sortilin‐related receptor 1; TMAO, trimethylamine N‐oxide; TREM2, triggering receptor expressed on myeloid cells 2.

### 2.2 Limitations of current single‐target therapeutic strategies

The complexity of AD pathogenesis has spurred the development of therapies aimed at individual pathways, yet clinical success has remained limited.

#### 2.2.1 Anti‐Aβ therapies

Monoclonal antibodies such as aducanumab and lecanemab represent notable advances in directly targeting amyloid pathology. In the EMERGE trial, aducanumab significantly reduced cerebral amyloid plaques, yet its cognitive benefits were modest and failed to reach clinical significance, alongside a high incidence of amyloid‐related imaging abnormalities (ARIA).^61^ Lecanemab, evaluated in a phase III study, demonstrated more pronounced effects—significant amyloid reduction and slower cognitive decline in patients with early AD.^62^ However, its efficacy remains confined to early disease stages, and ARIA‐related safety concerns persist.^63^ These outcomes underscore the difficulty in achieving meaningful clinical improvement by targeting Aβ after extensive downstream pathology—such as tau tangles and neurodegeneration—has already developed.

#### 2.2.2 Tau‐targeted therapies

Tau‐directed strategies include antibodies such as gosuranemab, designed to inhibit the cell‐to‐cell spread of pathogenic tau by targeting its extracellular form. Although early studies showed that gosuranemab reduced Cerebrospinal Fluid (CSF) levels of phosphorylated tau, it failed to slow cognitive decline in the phase II TANGO study.^64^, ^65^ This setback highlights a major obstacle: effectively targeting intracellular neurofibrillary tangles with large‐molecule antibodies remain challenging due to their limited BBB and poor access to the primary site of tau pathology.^36^

#### 2.2.3 Other pathway‐specific drugs

Therapies targeting neuroinflammation (e.g., TREM2 activators such as AL002),^66^, ^67^ mitochondria dysfunction (e.g., Fis1 inhibitors like P110)^68^, ^69^ or metabolism pathways (e.g., intranasal insulin)^70^ have shown preclinical promise but face obstacles in clinical translation, including insufficient efficacy in pivotal trials, safety issues or inadequate BBB penetration.

Collectively, experience with these single‐target agents underscores a key insight: modulating one node within AD's interconnected pathogenic network is often insufficient. This reality emphasises the need for multi‐target strategies capable of simultaneously addressing several core disease mechanisms.

### 2.3 From mono‐targeting limitations to engineered exosomes as an integrated platform

The well‐documented limitations of single‐target pharmacotherapy—characterised by modest efficacy and an inability to meaningfully alter disease progression—highlight a fundamental mismatch between mono‐mechanistic interventions and the complex, networked pathology of AD. This therapeutic disconnect has accelerated the development of platforms capable of simultaneously targeting multiple pathological pathways. Among these, synthetic nanocarriers and cell‐based therapies have emerged as two leading alternative strategies, each offering unique advantages as well as inherent limitations.

A critical evaluation of the data in Table 2 enables a systematic comparison across platforms, revealing a distinct trade‐off between functionality, safety and translational capacity. Synthetic nanocarriers (e.g., PLGA nanoparticles, liposomes) possess a high drug‐loading capacity and offer considerable engineering flexibility.^71^, ^72^ While their penetration across the BBB can be significantly enhanced through surface modification,^73^, ^74^ they often lack intrinsic biological targeting specificity and may provoke innate immune reactions. In contrast, cell therapies, such as mesenchymal stem cells (MSCs) transplantation, provide potent multi‐mechanistic synergy via the secretion of diverse neuroprotective factors.^75^, ^76^, ^77^ However, they face challenges including low targeting precision, the inherent risk of tumorigenicity associated with live cell administration, and complex manufacturing and logistical requirements.

**TABLE 2:** Comparative analysis of therapeutic strategies for Alzheimer's disease (AD).

Comparison dimension	Monotargeted drugs (e.g., donepezil, lecanemab)	Nanocarriers (e.g., PLGA NPs, liposomes)	Cell therapies (e.g., MSCs transplantation)	Engineered exosomes
Representative technology	Donepezil, memantine, lecanemab, donanemab	PLGA nanoparticles, liposomes	MSC intravenous transplantation, iPSC‐derived neurons	MSC exosomes, RVG‐modified exosomes
Core mechanism	Single‐target modulation^78^, ^79^ (e.g., AChE inhibition, Aβ clearance)	Synthetic drug delivery vehicle^71^, ^72^ (passive/active targeting)	Paracrine secretion of trophic factors (e.g., BDNF, VEGF); cell replacement^75^, ^76^	Multi‐target activity: drug delivery, Aβ clearance, anti‐inflammation, neuroprotection^80^
Target specificity	High^81^ (designed for single molecular target)	Engineered for high specificity via surface ligands^82^	Low^83^ (broad systemic effects)	Moderate to high^84^ (enhance able via engineering)
Multi‐mechanistic synergy	Limited^85^ (single mechanism of action)	Dependent on payload; capable of co‐delivery^74^	Strong^77^ (anti‐inflammatory, neurodegenerative, Aβ clearance)	Strong synergistic potential^80^ (synaptic repair, multi‐pathway modulation)
BBB penetration efficiency	Low^11^ (most require structural modification or facilitation to cross the BBB)	Moderate^73^ (enhance able via functionalisation)	Low^86^ (limited innate tropism; engineered homing under study)	High^87^ (native BBB crossing; modifiable by source/engineering)
Drug loading capacity	Not applicable (not a delivery system)	High (high encapsulation efficiency for various cargos)	Low and heterogeneous (dependent on cellular secretion)	Moderate to high (loading efficiency and capacity vary with method and cargo)
Period of effectiveness	Short to medium term^88^ (requires repeated administration)	Short term^89^ (controlled release over days to weeks)	Long term^90^ (sustained benefit post‐engraftment)	Medium term^91^ (weeks in preclinical models)
Safety risks	Common adverse effects (nausea, dizziness, hallucinations)	Innate immunogenicity, complement activation	Immunogenicity, thromboembolism, tumourigenic risk	Favourable in preclinical studies; low immunogenicity due to endogenous origin
Engineering capability	Limited (chemical modification for BBB)	Surface functionalisation, polymer optimisation	Genetic editing (e.g., BDNF overexpression)	Highly engineerable (miRNA editing, ligand modification)
Clinical translation Stage	Approved or filed for approval^92^	Primarily preclinical^93^ (e.g., Curcumin‐PLGA NPs)	Phase I/II trials (NCT03117738)	Predominantly preclinical research; early clinical trials initiated (NCT04388982)
Cost and economic feasibility	High (drug cost, IV infusion, frequent MRI monitoring)	Generally lower (cargo‐dependent)	High (cell preparation, infusion procedures)	Currently high production cost; future reduction potential via automation

Abbreviations: Aβ, amyloid‐beta; AChE, acetylcholinesterase; BBB, blood–brain barrier; BDNF, brain‐derived neurotrophic factor; iPSC, induced pluripotent stem cell; IV, intravenous; MSC, mesenchymal stromal cell; NP, nanoparticle; PLGA, poly(lactic‐co‐glycolic acid); RVG, rabies virus glycoprotein; VEGF, vascular endothelial growth factor.

In this evolving landscape, engineered exosomes have emerged as an integrative platform that combines advantageous features from both domains. By nature, they exhibit an innate ability to interact with and cross the BBB, a critical feature for CNS therapeutics.^87^ They support moderate‐to‐high drug loading and demonstrate intrinsic multi‐target functionality—including Aβ clearance, anti‐inflammatory activity and synaptic repair.^80^, ^84^ Furthermore, their safety profile appears favourable: preliminary studies suggest low immunogenic potential, and, as acellular entities, they circumvent the tumorigenesis risk associated with their parent cells.

In summary, this chapter has outlined the shift from single‐target drugs towards multi‐target platforms, motivated by the intricate and interconnected nature of AD pathogenesis. Engineered exosomes represent a convergent strategy that merges the engineerability of synthetic nanocarriers with the innate biological functionality of cell‐derived systems. Although their path to clinical application has faced numerous challenges, their unique ability to simultaneously target multiple disease mechanisms—coupled with favourable safety and drug delivery profiles—positions them as strong candidates for next‐generation AD therapies. The following sections will examine specific engineering strategies designed to realise this potential in the context of AD.

## 3 EXOSOME BIOLOGY: A FOUNDATION FOR ENGINEERED MULTI‐TARGET THERAPY

The native biology of exosomes—specifically their formation pathway and structural architecture—constitutes a unique and adaptable foundation for engineering multi‐target therapeutic strategies. A thorough understanding of this biological basis is therefore essential for appreciating their full therapeutic potential.

### 3.1 Biogenesis as a platform for drug loading

**FIGURE 1:**
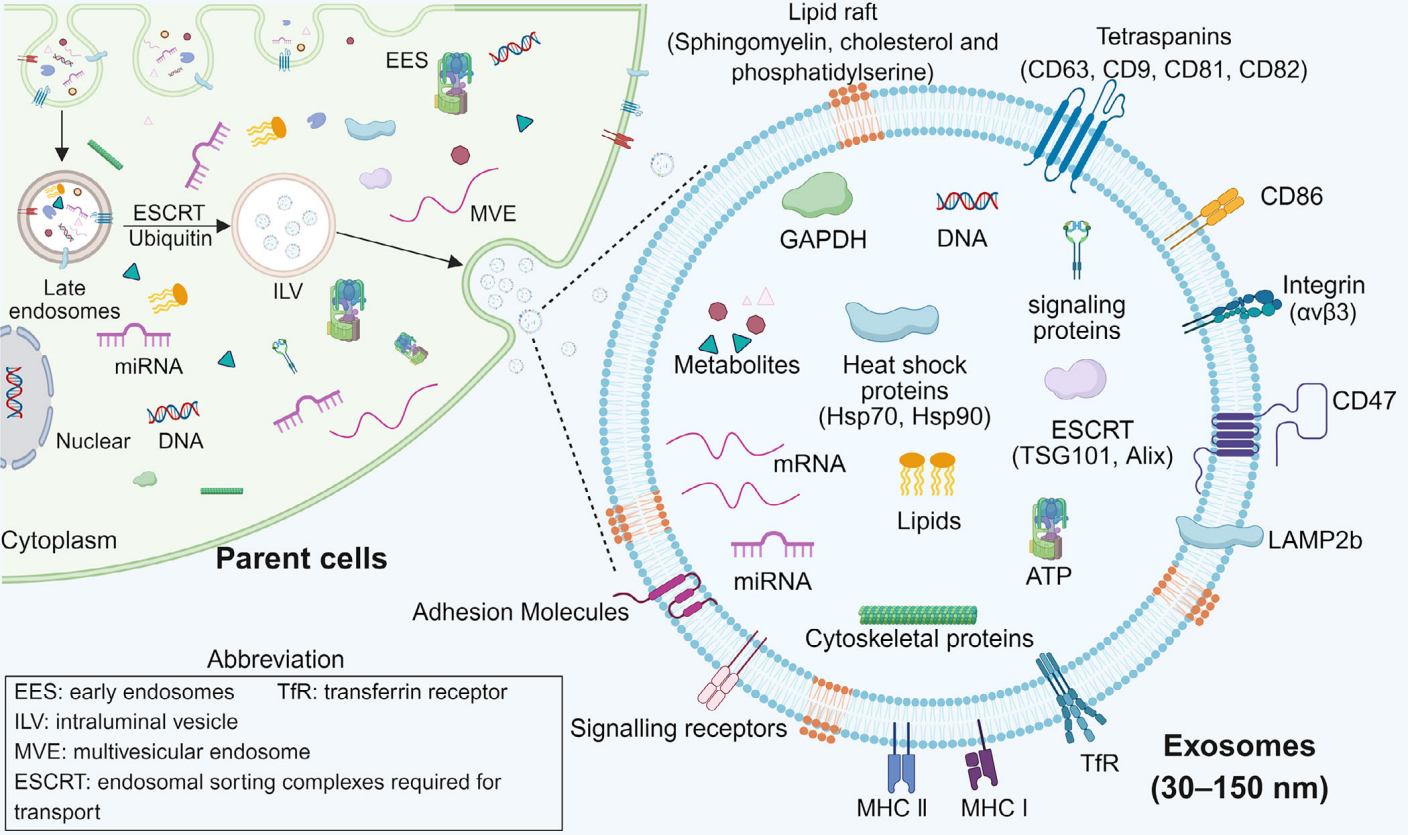
Biosynthesis and architecture of exosomes. Exosomes are formed within multivesicular endosomes via the ESCRT pathway and then released. They have a complex structure containing multiple marker proteins, nucleic acids, lipid rafts and cytoskeletal proteins.

The endosomal pathway of exosome biogenesis offers inherent mechanisms for loading diverse therapeutic cargo. The process begins with the endocytosis of the cell membrane, leading to the formation of early endosomes.^94^ Subsequently, these early endosomes mature into late endosomes, during which their membranes invaginate to form intraluminal vesicles (ILVs). This ILV formation, driven by mechanisms such as the ESCRT complex, serves as the primary packaging step, encapsulating cytoplasmic proteins and nucleic acids within the vesicle lumen. Finally, upon fusion of the mature multivesicular body with the plasma membrane, these ILVs are released into the extracellular space as exosomes, which typically range from 30 to 150 nm in diameter.^95^ Figure 1 illustrates the exosome production process, along with key surface and internal features detailed in subsequent sections. A significant advantage of this system is that the endogenous packaging process can be co‐opted to load combination of therapeutic molecules (e.g., proteins, RNAs) simultaneously during exosome formation, thereby directly facilitating a multi‐target approach.^96^

### 3.2 Structure enabling multi‐targeting and delivery

The native architecture of exosomes is uniquely suited for complex delivery tasks. Their membrane is a lipid bilayer enriched with specific proteins that govern cellular interactions. Key surface components include: tetraspanins (e.g., CD63, CD9, CD81), which facilitate cellular uptake and can be engineered for targeted delivery; integrins, which mediate specific adhesion to recipient cells (e.g., αvβ3 integrin binding to ICAM‐1 to facilitate BBB penetration); and immunomodulatory proteins (e.g., MHC, CD47), which contribute to biocompatibility and immune evasion.^97^, ^98^ Internally, the exosome lumen carries a diverse payload of bioactive molecules. The combination of an engineered surface that can display targeting ligands and a spacious lumen capable of encapsulating multiple therapeutic agents makes exosomes an ideal platform for multi‐targeting strategies, enabling the simultaneous presentation of specific targeting peptides and the delivery of combination therapies directed at distinct pathological pathways.^95^

### 3.3 Comparative advantages for multi‐targeted therapy in AD

To systematically position engineered exosomes within the current therapeutic landscape for AD, we compare them in Table 3 against several nanocarrier platforms, including conventional liposomes,^99^ solid lipid nanoparticles (SLNs)^100^ and the emerging class of exosome–liposome hybrids.^101^ This comparative analysis extends beyond fundamental drug delivery metrics to encompass critical translational factors such as BBB penetration, targeting precision, immunogenicity and manufacturing scalability.^18^

**TABLE 3:** Comparative analysis of nanocarrier platforms for overcoming the blood‒brain barrier (BBB) in Alzheimer's disease therapy.

Performance metric	Exosomes	Conventional liposomes	Solid lipid nanoparticles	Exosome‒liposome hybrids
Drug loading efficiency	Moderate to low—methods under exploration; reproducibility challenging^102^	High—mature technology for hydrophilic/hydrophobic drugs^103^	Moderate—limited by solid lipid matrix; drug leakage possible^104^	High—inherits structural advantages of liposomes^20^
BBB penetration capability	High—efficiently crosses via receptor‐mediated transcytosis^105^	Low—requires surface modification for limited penetration^106^	Needs modification to improve—small size and lipophilic nature allow some penetration^107^	Higher than single system—combines innate exosomal penetration with engineered modifications^20^
Targeting ability	High (inherent)—natural homing to pathological tissues^108^	Low (requires engineering)—needs conjugation of antibodies/ligands^109^	Low (requires engineering)—similar to liposomes, needs functionalisation^110^	Very high (inherent + engineered)—natural targeting + precision engineering^111^
Immunogenicity	Very low—endogenous origin; excellent biocompatibility^112^	Moderate to high—exogenous entities may trigger immune recognition^109^	Low to moderate—generally biocompatible but may elicit mild responses^113^	Low—exosomal membrane provides immune ‘camouflage’^101^
Circulation half‐life	Long—surface proteins inhibit macrophage phagocytosis^114^	Short (extendable)—prone to reticuloendothelial system clearance; often PEGylated^109^	Moderate (extendable)—susceptible to clearance; modifiable^115^	Long—presumed to inherit exosomal stability; validation needed^20^
Production and standardisation	Challenging—complex extraction; low yield; difficult scale‐up^116^	Mature—well‐established processes; clinically approved^109^	Relatively mature—established and scalable technology^117^	Complex, under development—standardisation and scalability remain challenges^118^

Abbreviation: PEGylated, polyethylene glycol‐modified.

Our analysis reveals a distinctive profile for engineered exosomes. They exhibit inherently high BBB penetration, mediated primarily by receptor‐mediated transcytosis (RMT),^18^ alongside an intrinsic targeting capability conferred by their natural homing properties—features that conventional liposomes and SLNs largely lack without extensive engineering.^17^ Furthermore, their endogenous origin imparts very low immunogenicity and a prolonged circulation half‐life, which collectively reduce clearance risks and enhance therapeutic durability. Notably, their structural versatility facilitates a multi‐target mechanism of action, enabling simultaneous drug delivery, Aβ clearance and neuroprotection—a clear advantage over single‐target therapeutic modalities.^119^

Exosome–liposome hybrids represent a promising integrative strategy, effectively combining the high drug‐loading capacity and synthetic controllability of liposomes with the bioinspired trafficking and low immunogenicity of exosomes.^111^, ^120^ However, challenges remain in achieving efficient drug loading and scalable production for both native and hybrid exosomal systems—areas where conventional liposomes and SLNs currently maintain a mature advantage. Despite these limitations, engineered exosomes and their hybrid derivatives constitute a transformative platform capable of addressing the multifactorial pathology of AD by leveraging their unique biological properties.^121^, ^122^

Notwithstanding the superior performance of exosome‒liposome hybrids in certain metrics—such as drug loading and BBB penetration—several rationales support the continued and parallel development of unmodified engineered exosomes. First, from a regulatory and clinical translation standpoint, the pathway for biological exosomes is likely more clearly defined than that for synthetic–natural hybrid constructs, which introduce additional characterisation challenges.^123^ Second, the production of hybrid systems entails greater complexity and cost, potentially undermining the cost‐effectiveness required for broad therapeutic application.^116^ Finally, in therapeutic contexts where the primary goal is to augment or exploit innate exosomal biological signalling—rather than to maximise synthetic drug payload—minimally modified exosomes may offer a more optimal balance of efficacy, safety and manufacturing feasibility.^124^ Thus, the selection between native exosomes and hybrid platforms should be guided by specific therapeutic objectives, with unmodified or minimally modified exosomes continuing to represent a viable and strategically sound option for diverse clinical applications.

## 4 FUNCTIONALLY DRIVEN ENGINEERING STRATEGIES FOR EXOSOMES

**FIGURE 2:**
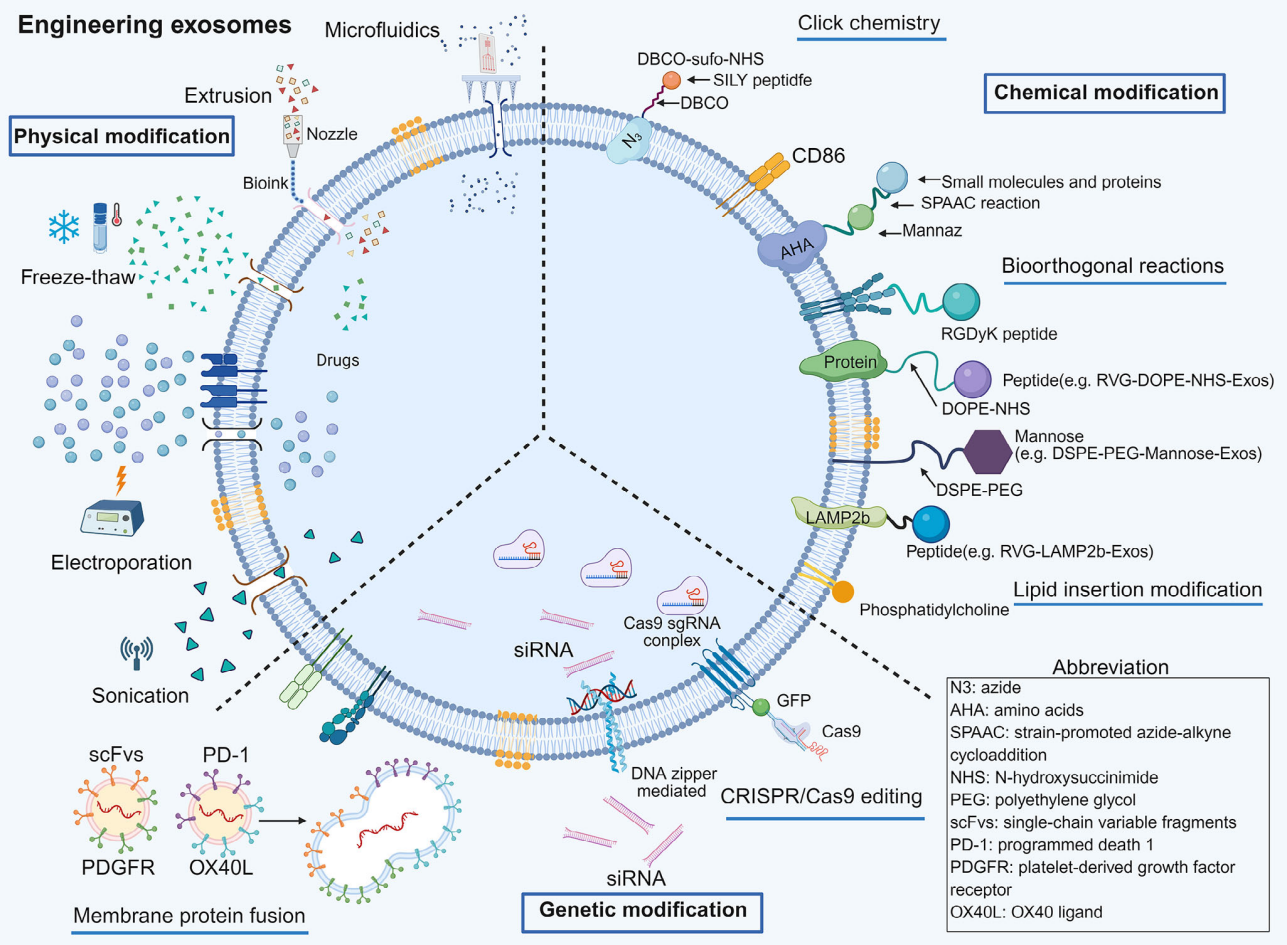
Strategies for engineering exosomes with functional molecules. This schematic summarises various approaches for conjugating small molecules, proteins, peptides and nucleic acids to exosomes. Key methodologies illustrated include: physical techniques such as extrusion, freeze‒thaw, electroporation and ultrasonication; chemical modifications using lipid insertion (e.g., DSPE‐PEG‐mannose) and bioorthogonal reactions (e.g., strain‐promoted azide‐alkyne cycloaddition [SPAAC]); genetic engineering via membrane protein fusion (e.g., LAMP2b‐RVG) or clustered regularly interspaced short palindromic repeats/CRISPR‐associated protein 9 (CRISPR/Cas9) editing; and other methods such as DNA zipper‐mediated assembly. These strategies enable the functionalisation of exosomes to improve drug loading and targeting specificity for therapeutic applications.

Figure 2 illustrates diverse engineering strategies for modifying exosomes, encompassing physical, chemical and genetic approaches. These methods are pivotal for augmenting the functionality and targeting efficiency of exosomes.

### 4.1 Physical modification for enhanced drug loading

Physical methods employ external forces to transiently disrupt the integrity of the exosomal membrane, thereby facilitating the encapsulation of therapeutic cargo. While generally straightforward, these techniques can potentially compromise membrane stability.

#### 4.1.1 Sonication and electroporation

Sonication utilises pulsed sound waves to induce cavitation effects, generating transient pores in the exosomal membrane that promote the entry of small‐molecule drugs.^125^, ^126^ Studies have demonstrated that sonicated exosomes significantly enhance drug delivery to ischaemic brain regions without inflicting healthy tissue.^127^ Electroporation, which applies high‐voltage electric fields to create reversible pores, is particularly suitable for loading large nucleic acids such as siRNA and miRNA.^128^, ^129^ For example, this technique has been successfully employed to load therapeutic siShn3 into BT‐Exos to enhanced osteogenic activity,^130^ and to encapsulate miR‐124 into MSC‐derived RVG‐Exos for targeted delivery to the ischaemic brain.^131^ However, careful optimisation is critical, as suboptimal conditions can lead to nucleic acid aggregation or membrane damage. Emerging techniques, including cell nanoporation and nanosecond pulse electroporation, have demonstrated improved loading efficiency.^132^, ^133^

#### 4.1.2 Freeze‒thaw cycles and extrusion

The freeze‒thaw method entails repeated cycles of freezing and thawing. During this process, the formation and subsequent rupture of ice crystals generate fissures in the exosomal membrane, enabling drug encapsulation. In contrast to sonication, this method has shown superior loading efficiency and better preservation of membrane integrity for small molecules such as methotrexate.^134^, ^135^ It is also effective for creating hybrid exosome‒liposome delivery systems.^111^

Extrusion involves forcing a mixture of exosomes and therapeutic agents through nano‐porous membranes under mechanical stress, facilitating forced fusion and cargo loading.^136^ While this method is utilised for drug loading, it is also applied to produce uniform exosome‐mimetics.^137^ A notable drawback is the potential alteration of native exosome properties due to the high shear forces involved.

#### 4.1.3 Microfluidic technology

Microfluidic technology affords precise control over fluids at the microscale, achieving high‐throughput exosome loading via the application of shear stress or pressure.^138^ A significant advantage lies in its potential for integration, allowing for the combination of purification and loading processes within a single device.^139^ For instance, a platform integrating microfluidics with electrospray and freezing technology has been developed to create intelligent microcarriers for the synergistic release of exosomes and drugs in the treatment of osteoarthritis.^140^

### 4.2 Chemical modification for improved stability and targeting

Chemical methods functionalise the exosome surface through covalent or non‐covalent interactions. These approaches are primarily employed to confer targeting capabilities or enhance stability, and can also be leveraged for efficient cargo loading via surface conjugation.

#### 4.2.1 Click chemistry and bioorthogonal reactions

**FIGURE 3:**
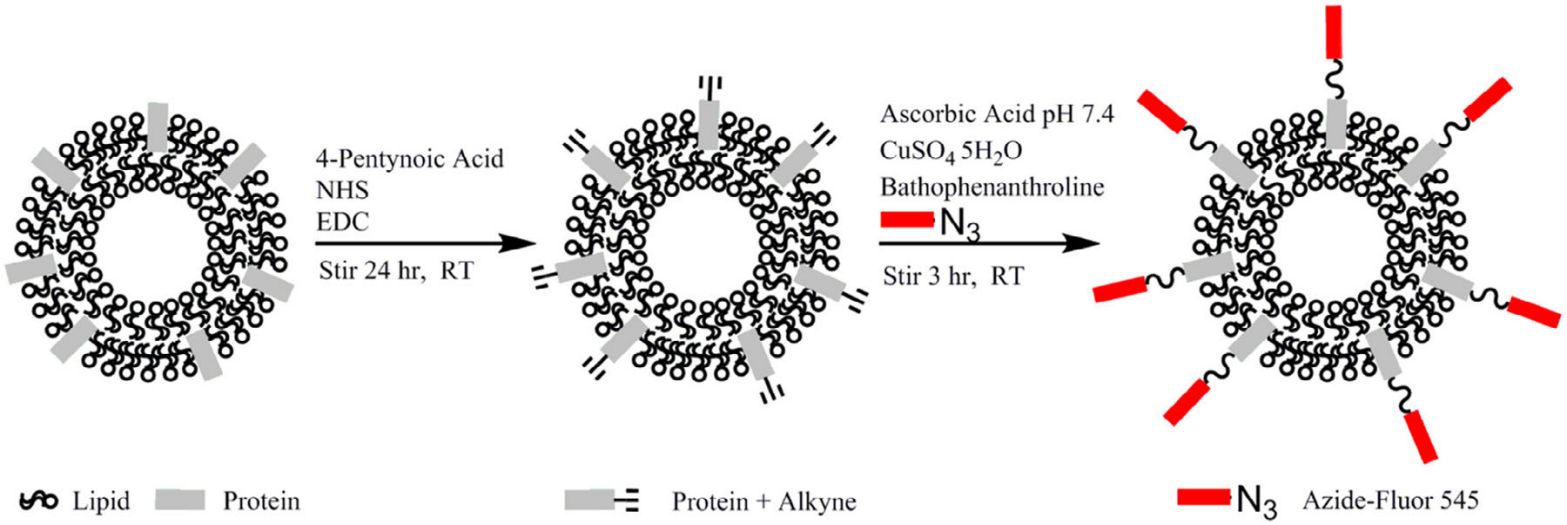
Strategy for exosome surface labelling through sequential chemical conjugation. Step 1: amine groups on the exosomal surface were activated and coupled with 4‐pentynoic acid to install terminal alkyne groups. Step 2: the alkyne‐tagged exosomes were subsequently labelled with Azide‐Fluor 545 via a Cu(I)‐catalysed cycloaddition, enabling fluorescence imaging.

Click chemistry, renowned for its high efficiency and specificity, has demonstrated significant utility in engineering exosome modification, particularly for targeted peptide conjugation, drug or gene loading, and multi‐functionalisation.^141^, ^142^, ^143^ As illustrated in Figure 3, a representative click chemistry reaction involves the reaction of 4‐pentynoic acid with N‐(3‐(dimethylamino)propyl)‐N′‐ethylcarbodiimide to form an NHS‐ester, which subsequently reacts with primary amines on proteins to introduce alkyne groups. Following this, a copper‐catalysed azide‐alkyne cycloaddition (CuAAC) is performed using an azide‐containing fluorophore (e.g., Azide‐Fluor 545) in the presence of a copper catalyst—generated in situ from CuSO_4_, ascorbic acid and the ligand bathophenanthroline—enabling fluorescent labelling of the protein. This highly efficient conjugation strategy has been successfully applied to exosome modification.^144^ For example, conjugation of the RGERPPR peptide to exosomes loaded with superparamagnetic iron oxide nanoparticles (SPIONs) and curcumin via click chemistry yielded constructs capable of targeted glioma imaging and therapy.^145^ Similarly, the attachment of a SILY peptide enhanced exosome retention within the extracellular matrix.^146^

**FIGURE 4:**
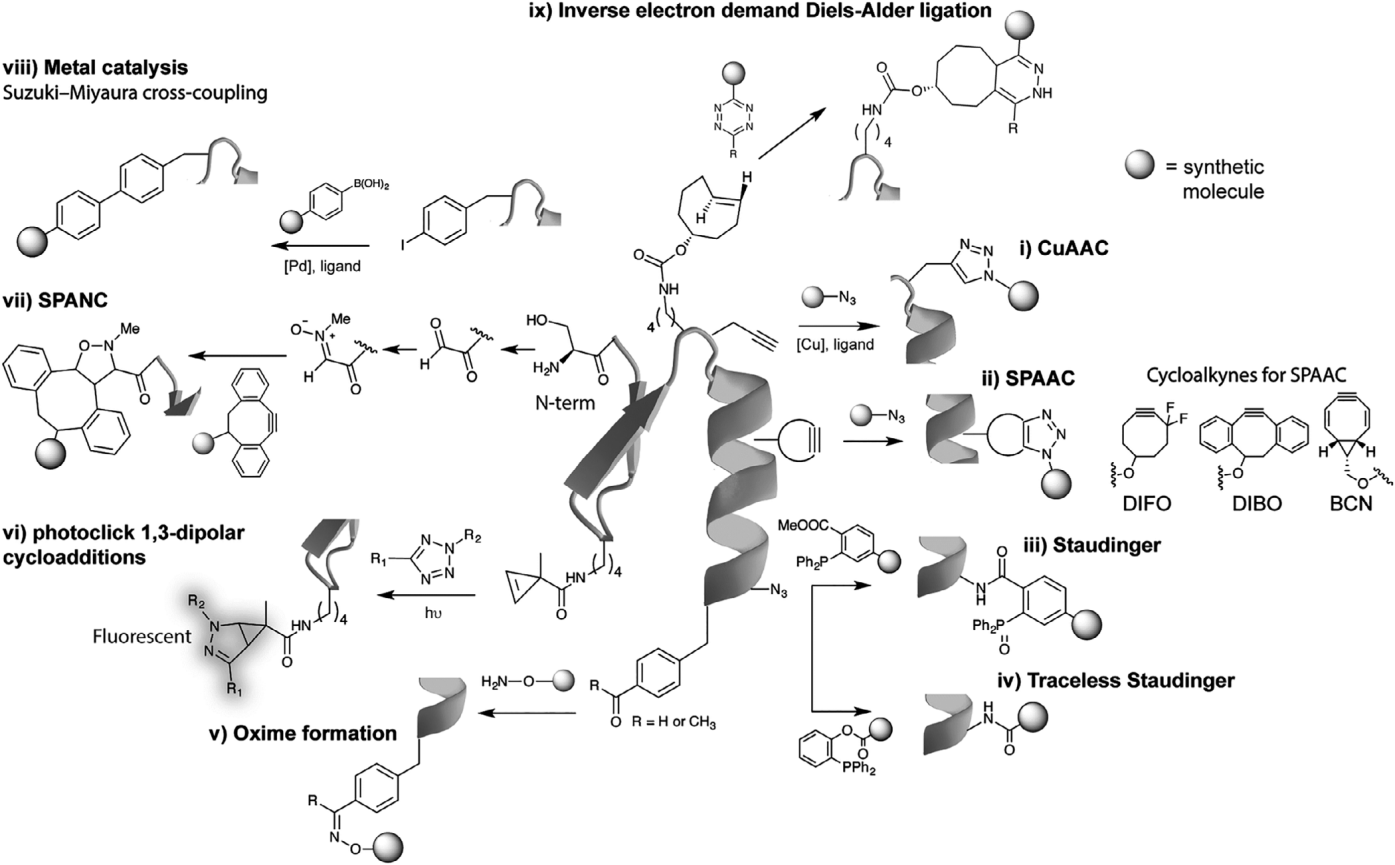
Representative examples of bioorthogonal reactions for protein modification. This schematic compares the mechanisms and representative reactants of several prominent bioorthogonal reactions used for site‐specific protein functionalisation. (i) Copper‐catalysed azide‐alkyne cycloaddition (CuAAC): copper‐catalysed cycloaddition between an azide and a terminal alkyne. (ii) Strain‐promoted azide‐alkyne cycloaddition (SPAAC): metal‐free, strain‐promoted cycloaddition between an azide and a cyclooctyne. (iii) Staudinger: reaction between an azide and a phosphine to form an amide bond. (iv) Traceless Staudinger: a variant that leaves no residual atoms in the final product. (v) Oxime formation: condensation between an aminooxy group and a carbonyl (aldehyde or ketone). (vi) Photoclick 1,3‐dipolar cycloadditions: light‐triggered reactions, such as between tetrazoles and alkenes. (vii) SPANC: strain‐promoted cycloaddition between a nitrone and a cyclooctyne. (viii) Metal catalysis: example shown is the Suzuki‐Miyaura cross‐coupling for C‒C bond formation. Protein residues are shaded in grey, with synthetic molecules represented as circular icons.

For in vivo applications, bioorthogonal reactions (e.g., copper‐free azide‐alkyne cycloaddition) are preferred due to their superior biocompatibility. As shown in Figure 4, bioorthogonal chemistry encompasses a range of highly specific reactions that proceed without interfering with biological processes. Representative examples include the strain‐promoted azide‐alkyne cycloaddition and the inverse electron demand Diels–Alder reaction, among others, which provide powerful tools for bioconjugation.^147^ Bioorthogonal chemistry has emerged as a transformative tool in the biomedical research field with its high specificity, rapid reactivity, good biocompatibility and modular design flexibility.^148^, ^149^, ^150^ A common strategy involves metabolic labelling of parent cells with azide‐containing moieties, enabling subsequent specific conjugation on the secreted exosomes for tracking or targeted delivery applications.^151^, ^152^

#### 4.2.2 Lipid insertion

Lipid insertion represents a straightforward and effective non‐covalent strategy. This approach capitalises on the amphiphilic nature of lipid molecules to associate with the exosomal membrane via hydrophobic interaction or electrostatic adsorption, thereby modulating membrane fluidity, targeting specificity and cellular uptake efficiency.^153^ Amphiphilic lipid derivatives (e.g., 1,2‐distearoyl‐sn‐glycero‐3‐phosphoethanolamine‐polyethylene glycol [DSPE‐PEG]) can insert their hydrophobic tails into the exosomal membrane lipid bilayer, exposing hydrophilic functional groups (e.g., mannose, targeting ligands) on the surface. Studies indicate that the insertion of phosphatidylcholine molecules can nearly double the cellular uptake efficiency of exosomes, likely by enhancing membrane fusogenicity or facilitating specific endocytic pathways.^154^ Furthermore, the insertion of DSPE‐PEG‐mannose has been utilised to achieve targeted delivery to microglia.^155^ The stability of this modification is contingent upon the strength of the hydrophobic interactions with the membrane.

### 4.3 Genetic engineering of parent cells for innate modification

Genetic engineering modifies parent cells to inherently tailor exosome composition and function, resulting in stable and heritable modifications.

#### 4.3.1 Membrane protein fusion

This strategy involves genetically fusing a protein of interest (e.g., a targeting scFv antibody, an immunomodulatory protein) to a transmembrane domain or terminal region of an abundant exosomal membrane protein (e.g., CD9, CD63). When expressed in parent cells (e.g., HEK293 cells), the resultant fusion protein is naturally sorted into the exosomal membrane. This methodology has been employed to create multifunctional exosomes capable of displaying multiple proteins simultaneously.^156^ Alternatively, DNA zipper‐mediated membrane fusion can be employed to hybridise exosomes with liposomes for efficient siRNA loading.^157^

#### 4.3.2 CRISPR/Cas9 editing technology

Clustered regularly interspaced short palindromic repeats/CRISPR‐associated protein 9 (CRISPR/Cas9) technology can be utilised not only to edit the genome of parent cells to alter exosome biology but also to be loaded as a therapeutic cargo into exosomes. Once ingenious approach leverages high‐affinity protein interactions (e.g., between GFP and its nanobody) to enrich Cas9 protein into exosomes engineered to express a CD63‐GFP fusion protein.^21^ Other strategies involve engineering exosomal membrane proteins (e.g., CD9‐HuR) to possess RNA‐binding capacity, enabling the enrichment of specific miRNAs or sgRNAs.^158^ Additionally, displaying viral envelope proteins such as VSV‐G on exosomes can enhance cellular fusion and the delivery efficiency of CRISPR/Cas9 components.^159^ By integrating these approaches, researchers can tailor exosomes for diverse applications, ranging from drug delivery to gene therapy.

The choice of an appropriate engineering strategy depends on factors such as the nature of the cargo (size, hydrophilicity), the required targeting precision, the necessity to preserve innate exosome properties, and scalability considerations. As summarised in Table 4, each method possesses distinct advantages and limitations. Physical methods are versatile but may compromise membrane integrity; chemical approaches offer precision but often require pre‐modification of molecules; genetic engineering provides stable, innate modifications but entails greater technical complexity. Future development will likely involve combining these strategies (e.g., genetic pre‐modification followed by chemical conjugation or physical loading) to create next‐generation, smart exosome therapeutics.

**TABLE 4:** Comparative analysis of exosome engineering strategies.

Strategy	Sub‐method	Mechanism	Key advantages	Key limitations	Ideal application context	References
Physical	Sonication	Ultrasound‐induced cavitation creates transient pores	Simple, applicable to various drugs	Low drug loading efficiency (<10%); potential membrane damage	Loading small‐molecule drugs where mild membrane integrity loss is acceptable	^126^, ^160^, ^161^
	Electroporation	Electrical pulses generate reversible pores	Efficient for large molecules (e.g., nucleic acids)	Risk of cargo aggregation/denaturation; sensitive to buffer conditions	Loading siRNA, miRNA, plasmid DNA	^128^, ^162^
	Freeze‒thaw	Membrane disruption and resealing via ice crystal formation	Simple, low equipment needs, relatively mild	Low drug loading efficiency (5%‒15%); requires strict protocol control	Loading small drugs; creating hybrid exosome‒liposome systems	^134^, ^135^, ^158^
	Extrusion	Mechanical forcing through nanopores induces fusion/loading	Produces homogeneous preparations	High shear stress may alternative properties	Generating exosome mimetics; loading membrane‐anchored peptides	^137^, ^163^
	Microfluidics	Shear stress or acoustic wave effects in microchannels promote membrane permeability	High throughput; precise control; easily integrated	High equipment cost; potential channel clogging	Exosome isolation and integrated processing, such as drug loading via acoustic microfluidics	^139^, ^161^, ^164^
Chemical	Click chemistry	High efficiency, specific covalent conjugation (e.g., azide‐alkyne)	High specificity, mild conditions, modular	Requires pre‐functionalisation; copper catalysts may affect the biological activity of exosomes	Covalent conjugation of targeting peptides, tags or drug/gene loading on the surface	^143^, ^144^
	Bioorthogonal reaction	Click chemistry compatible with living systems	Biocompatible, suitable for in vivo labelling/targeting	Requires metabolic labelling; multi‐step process	In vivo tracking and targeting via pre‐labelling of parent cells	^147^, ^165^
	Lipid insertion	Hydrophobic insertion of amphiphilic lipids into the membrane	Simple, no special equipment, minimal membrane disturbance	Stability may be affected by in vivo environment (e.g., by protein corona)	Rapidly imparting targeting ability (e.g., using DSPE‐PEG‐ligands)	^153^, ^166^
Genetic engineering	Membrane protein fusion	Expression of fusion proteins (target protein‐transmembrane domain) in parent cells	Stable modification, innate to exosome, high yield	Technically complex, time consuming, may affect cell/exosome biology	Stable expression of targeting proteins or therapeutic proteins on exosomes; creating hybrid exosome‒liposome systems for siRNA loading	^111^, ^157^
	CRISPR/Cas9	Editing parent cell genome or loading Cas9/sgRNA as cargo	Fundamentally alters exosome function; high potential	Technically complex, off‐target risks, safety/ethical concerns for therapy	Basic research and developing advanced smart exosome therapeutics	^21^, ^167^

Abbreviations: DSPE‐PEG, 1,2‐distearoyl‐sn‐glycero‐3‐phosphoethanolamine‐polyethylene glycol; CRISPR/Cas9, clustered regularly interspaced short palindromic repeats/CRISPR‐associated protein 9; sgRNA, single‐guide RNA.

## 5 APPLICATIONS OF ENGINEERED EXOSOMES IN TREATMENT OF AD

The application of engineered exosomes in AD therapy has progressed through numerous innovative strategies designed to overcome the therapeutic challenges posed by the disease's complex pathophysiology. This section methodically evaluates these advancements, following a logical progression: (1) it first addresses the fundamental challenge of delivering therapeutics across the BBB to the site of pathology; (2) next, it explores engineering strategies for achieving precise, spatiotemporally controlled release of therapeutic cargo within the AD microenvironment; and (3) finally, it examines multi‐target synergistic therapies that concurrently confront diverse pathological features of AD to enhanced therapeutic outcomes (3). Together, these integrated strategies represent a rapidly advancing portfolio of exosome‐based technologies poised to address the intricate nature of AD.

### 5.1 Engineering exosomes for enhanced brain delivery and biodistribution

Achieving sufficient delivery of therapeutics to the brain parenchyma is a critical first step. Strategies in this domain focus on either engineering exosomes themselves for targeted delivery or employing innovative administration routes to bypass or modulate the BBB.

#### 5.1.1 Ligand‐mediated brain‐targeted delivery

**FIGURE 5:**
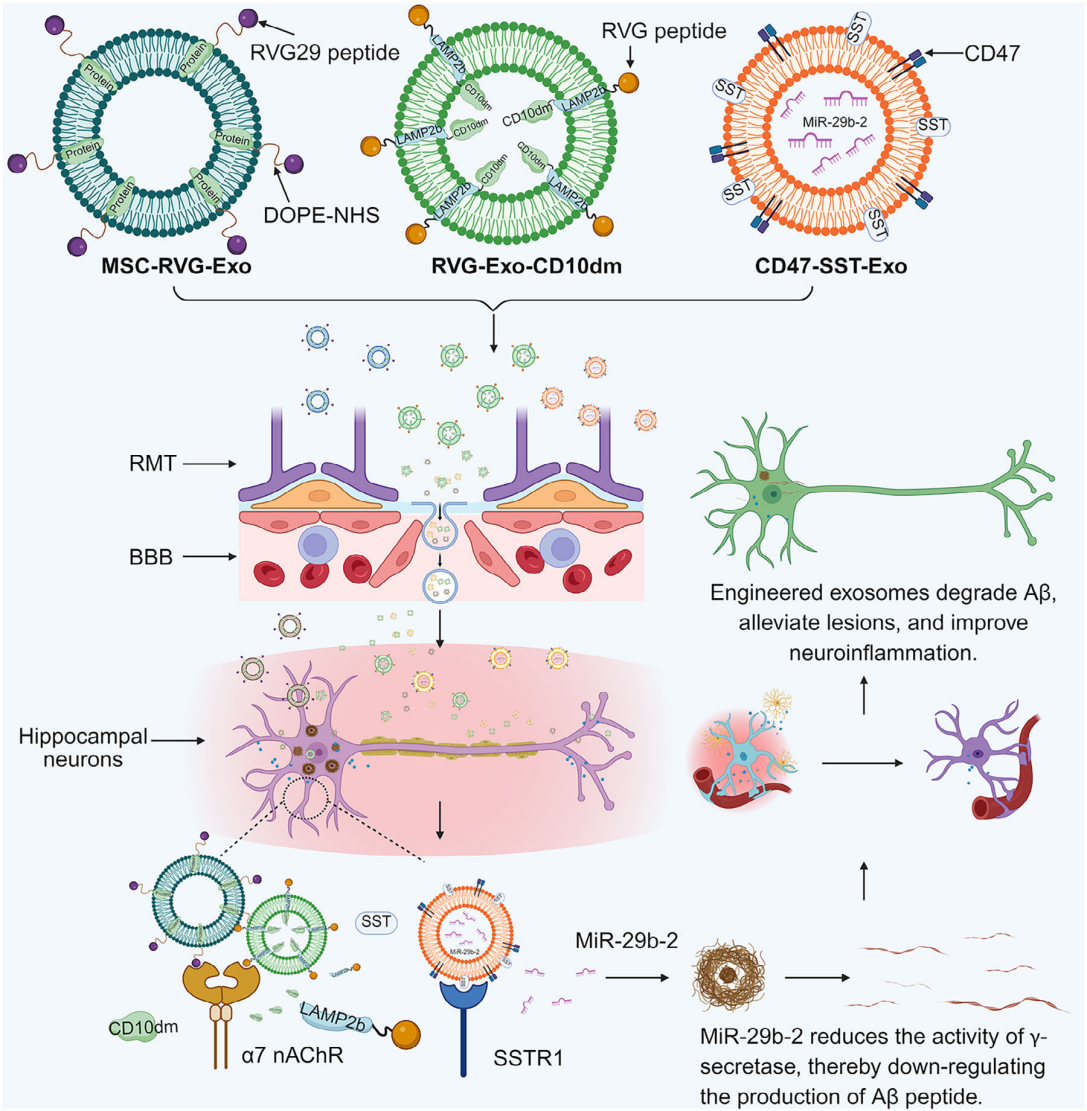
Targeted delivery of exosomes in the treatment of Alzheimer's disease (AD). By modifying targeting molecules such as RVG, CD10dm, CD47 and SST, exosomes cross the blood‒brain barrier and target hippocampal neurons. MSC‐RVG‐Exo improves neurological function, RVG‐Exo‐CD10dm reduces inflammation and inhibits astrocyte activation, and CD47‐SST‐Exo downregulates amyloid‐beta (Aβ) production through miR‐29b‐2, collectively improving neuroinflammation and neuropathological damage.

An overview of these techniques is presented in Figure 5. A primary strategy involves functionalising the exosome surface with targeting ligands. The RVG peptide, which binds specifically to nicotinic acetylcholine receptors (nAChR) on neurons, is widely employed. When conjugated to the exosome membrane, RVG29 enables efficient BBB transit via RMT, facilitating the targeted delivery of therapeutic molecules to key AD pathological regions such as the cerebral cortex and hippocampus.^168^, ^169^ For example, Cui et al. isolated exosomes from MSCs culture supernatant and used a DOPE‐NHS linker to conjugate the RVG peptide, successfully generating brain‐targeted MSC‐RVG‐Exo. Their results demonstrated that MSC‐RVG‐Exo effectively target the cortex and hippocampus of AD mice, significantly improve learning and memory, reduce Aβ level and plaque deposition, modulated the balance of brain inflammatory factors and suppressed the astrocytes activation.^170^ In a separate approach, Yu's team employed genetic engineering to co‐transfect plasmids encoding an RVG peptide fused with lysosome‐associated membrane glycoprotein 2b (LAMP2b) and a plasmid encoding a mutated CD10 (CD10dm) into parental cells, producing engineered exosomes (RVG‐Exo‐CD10dm) displaying surface RVG and enriched CD10dm. These exosomes were internalised in an α7‐nAChR‐dependent manner, targeted the hippocampus in vivo, reduced Aβ40 levels in N2a cells, and created an anti‐inflammatory microenvironment in aged mice.^19^ Other targeting modalities include the work by Lin et al., who genetically engineered dendritic cells to secrete exosomes co‐expressing CD47—which extends circulation time by reducing monocyte‒macrophage system clearance—and somatostatin receptors (SST), which bind to hippocampal neurons. These exosomes were loaded with miR‐29b‐2 to reduces γ‐secretase activity via downregulation of *PSEN1*. In 3xTg AD mice, these CD47‐SST exosomes significantly reduced hippocampal Aβ deposition and improved spatial learning and memory without eliciting significant immune responses.^171^

#### 5.1.2 Innovative administration methods that bypass the BBB

Innovative administration methods that bypass the BBB are essential for improving biodistribution.

- *Intranasal administration*: this route enables drugs to bypass the BBB rapidly via the nasal mucosa, avoiding first‐pass metabolism and enhancing bioavailability. For instance, Peng et al. successfully loaded miR‐206‐3p antagomir into bone marrow mesenchymal stem cell‐derived exosomes (MSC‐EVs) via electroporation to construct engineered MSC‐EVs Anta. Nasal administration of MSC‐EVs Anta produced significant therapeutic effects in an AD mouse model.^172^ Specifically, the treatment effectively reduced miR‐206‐3p levels in the prefrontal cortex and hippocampus while upregulating BDNF expression, activating BDNF/TrkB signalling pathway, and enhancing the phosphorylation of TrkB, Akt, ERK and CREB. In another study, the same team encapsulated BACE1 siRNA and berberine into exosomes derived from RVG29‐modified umbilical cord mesenchymal stem cells (UC‐MSCs) to construct a multifunctional exosome (MsEVB@R/siRNA). This formulation enabled direct drug penetration across the BBB into the brain. Experimental results demonstrated that these multifunctional exosomes effectively targeted neuronal and BV2 cells, significantly improved cognitive function, reduced Aβ deposition, suppressed neuroinflammation and promoted neural repair in a 5xFAD mouse model.^173^ Similarly, Han's team administered their MAPLEX system (a dCas9‐DNMT3A complex targeting BACE1) via intranasal delivery to 5xFAD and 3xTg‐AD models. The nasally delivered MAPLEX system crossed the BBB, delivered its cargo to neuronal cells, promoted methylation of the BACE1 promoter, reduced BACE1 expression, decreased Aβ plaque load and ameliorated cognitive memory deficits.^174^
- *Focused ultrasound (FUS)‐assisted delivery*: ultrasound technology can transiently open the BBB to enhance therapeutic delivery. The application of ultrasound in AD treatment has become a research focus. Ultrasonic vibration can be utilised to load drugs into exosomes, while FUS technology concentrates acoustic energy on specific areas brain regions, temporarily disrupting the BBB to permit the passage of drugs, antibodies, stem cells and other therapeutic substances into the brain, thereby improving treatment efficacy.^127^ A study by Deng et al. shown that FUS‐stimulated astrocytes release more exosomes. When combined with FUS‐mediated BBB permeabilisation, exosomes effectively crossed the BBB and reduced amyloid plaques and Aβ immunostaining in the brains of APP/PS1 mice.^175^ Zhu et al. investigated the safety, efficacy and practicality of ultrasound‐targeted microbubble destruction (UTMD) for the dual delivery of Aβ antibodies and neural stem cells in an AD mouse model. Their findings indicated that UTMD successfully transported therapeutics across the BBB into the brains of AD mice, facilitating Aβ plaques clearance, enhancing BDNF production and restoring impaired neurological function.^176^ A recent clinical trial provided preliminary support, demonstrating that FUS combined with an anti‐Aβ antibody led to a greater reduction of Aβ in the targeted brain region compared to the contralateral area after 6 months in three AD patients.^177^

### 5.2 Engineering exosomes for spatiotemporally controlled release

**FIGURE 6:**
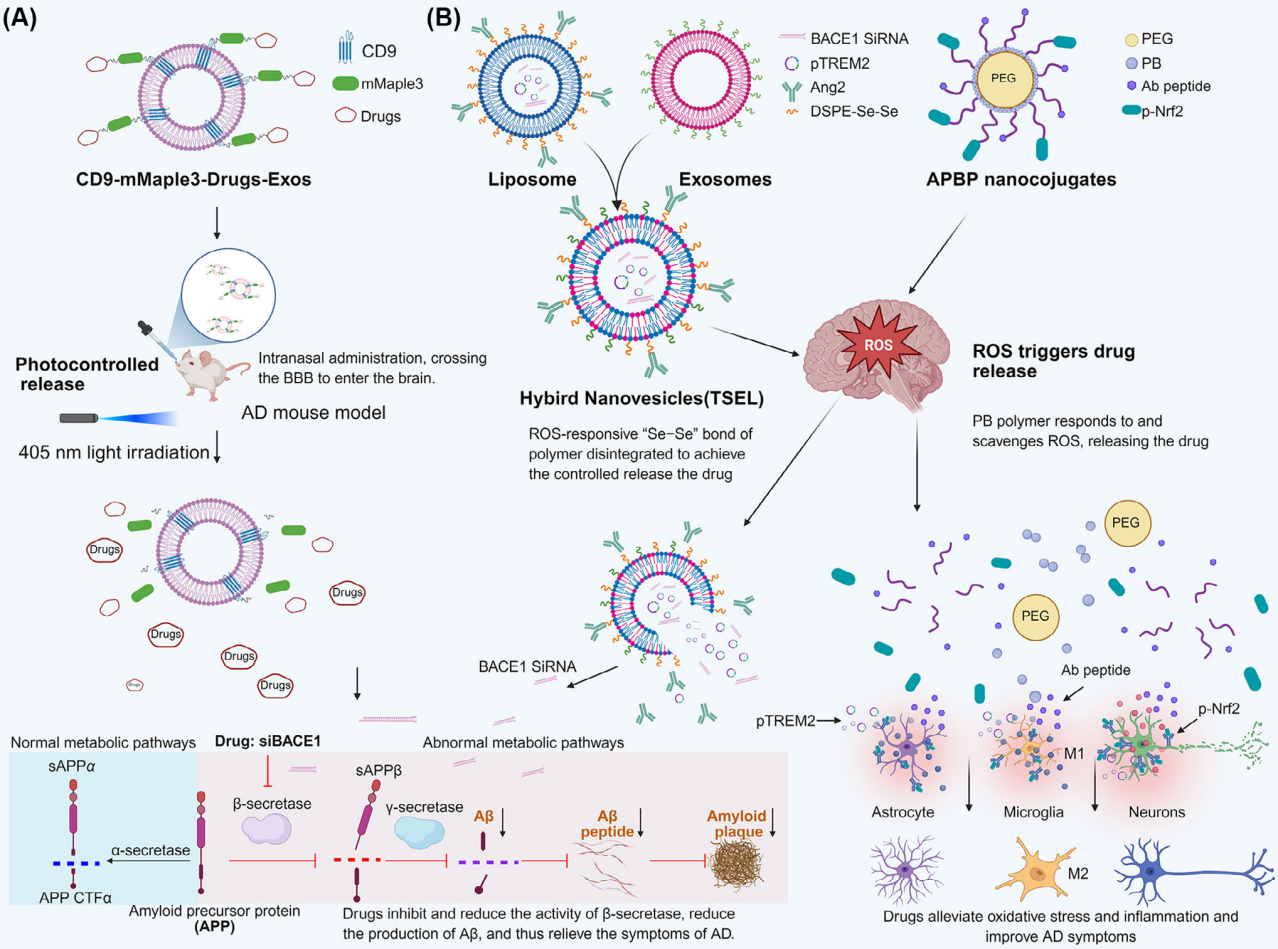
Intelligent responsive systems for Alzheimer's disease (AD) treatment. (A) MAPLEX system, which uses light to trigger drug release. CD9‐mMaple3‐Drugs‐Exos are engineered exosomes. Upon exposure to 405 nm light, the cargo proteins detach and enter cells, where siBACE1 reduces the production of amyloid‐beta (Aβ) by inhibiting β‐secretase activity. (B) TSEL and APBP systems, which respond to reactive oxygen species (ROS) in the AD brain. TSEL releases siBACE1 and pTREM2 via a ROS‐sensitive bond, reducing Aβ and modulating microglia. APBP releases p‐Nrf2 in ROS‐rich areas, activating antioxidant pathways and reducing inflammation.

Moving beyond passive delivery, engineered exosomes equipped with stimuli‐responsive cores (e.g., pH‐sensitive mental‐organic frameworks) and targeting ligands (e.g., RVG peptides) offer programmable platforms for the spatiotemporally controlled delivery of anti‐Aβ or anti‐tau therapeutics, addressing key limitations of native exosomes.^178^ These intelligent response systems are designed to regulate the release of exosomal cargo specifically within the AD microenvironment, thereby enhancing precision therapy. Figure 6 illustrates representative systems, including photocontrolled release (A) and reactive oxygen species (ROS)‐triggered release (B).

#### 5.2.1 Photocontrolled release systems

As illustrated in Figure 6A, Han's study developed an intracellular cargo protein delivery system based on mMaple3, a green‐to‐red light conversion fluorescent protein variant from the marine organism *Clavularia*.^174^, ^179^ A TagBFP‐mMaple3‐CD9 fusion protein was constructed by fusing mMaple3 with the exosomal membrane protein CD9. When overexpressed in HEK293T cells, this fusion protein localised to the cell plasma membrane and was subsequently loaded onto exosomes via CD9's inherent exosomal membrane targeting property, achieving efficient cargo proteins loading. Following exosome uptake by recipient cells, irradiation with 405 nm light triggers the photolysis of mMaple3, releasing the cargo protein from CD9 anchor to exert its function and induce diverse cellular events. In 3xTg AD and 5xFAD AD mouse models, this MAPLEX system, activated by 405 nm light, targeted the *BACE1* gene, induced its promoter DNA methylation, suppressed transcription, reduced β‐amyloid production, and ameliorated cognitive and memory deficits.^174^ In a related approach, Du's team created an intelligent, responsive biological interface termed the ferroelectric living interface for precise exosome secretion (LIFES). This system leverages distinctive topographical and electrical signals (piezoelectric and photopyroelectric effects), coupled with the sustained production of biologically active exosomes. The LIFES platform enables prolonged secretion (up to 192 h) and stage‐specific secretion (with an eightfold increase) of bioactive exosomes with tunable miRNA content.^180^ It demonstrates the capacity to significantly enhance neuronal development and connectivity, presenting a novel strategy for AD treatment.

#### 5.2.2 Microenvironment‐responsive release systems

Elevated levels of ROS in AD represent a key pathological feature and driver of disease progression.^181^ Wang et al. developed ROS‐responsive biomimetic exosome‒liposome hybrid nanovesicles (TSEL) to co‐deliver BACE1 siRNA (siBACE1) and a TREM2 plasmid (pTREM2). Leveraging the innate homing ability of exosomes and the angiopep‐2 polypeptide, these nanovesicles efficiently penetrate the BBB and accumulate in AD lesions. The upregulation of TREM2 expression promoted a shift in microglia from a pro‐inflammatory M1 phenotype to an anti‐inflammatory M2 phenotype, restoring their capacity to phagocytic Aβ and phenotype neural repair functions. Concurrently, siBACE1 reduced Aβ production, resulting in an enhanced therapeutic effect.^182^ Similarly, the Jiang team designed a ROS‐responsive dendrimer‒peptide conjugate (APBP) that targets the early inflammatory microenvironment of AD for multi‐target therapy. In the AD milieu, APBP responds to elevated ROS levels, releases its therapeutic peptide payload, inhibits neuroinflammation, restores neuronal antioxidant capacity and promotes Aβ clearance and glial cell phenotype normalisation. In the APP/PS1 mouse model, APBP reduced brain ROS levels, diminished Aβ deposition, mitigated glial cell activation and improved cognitive function, offering a potential avenue for early precision intervention and providing a technical foundation for multi‐target engineered exosome therapies.^183^ The multifactorial nature of AD necessitates therapeutic strategies that concurrently address its diverse pathological features, driving the development of multi‐target synergistic approaches that extend beyond smart targeting and release mechanisms.

### 5.3 Engineering exosomes for multi‐target synergistic therapies

Given the synergistic interplay among Aβ, tau, neuroinflammation, and oxidative stress, multi‐target synergistic treatments have emerged as a promising strategy to more effectively impede disease progression.

#### 5.3.1 Concurrent clearance of Aβ and tau pathologies

The Fe65 protein is a multi‐domain adaptor protein that can reduce Aβ42 production by promoting the α‐secretase cleavage of APP.^184^ Iyaswamy et al. generated Fe65‐engineered exosomes (Fe65‐Exo) by transfecting the Fe65 gene into the hippocampal neuronal cell line HT22 to achieve overexpression.^185^ They then loaded the autophagy inducer Corynoxine‐B (Cory‐B) into Fe65‐Exo via sonication and administered it intravenously to 5xFAD mice. Fe65‐Exo selectively bound to APP, facilitating targeted delivery Cory‐B to neurons with high APP expression. Cory‐B subsequently activated the phosphatidylinositol 3‐kinase catalytic subunit type 3 (PIK3C3) complex (composing BECN1, ATG5 and ATG7), promoting autophagosome formation. This mechanism effectively enhanced the encapsulation and degradation of both Aβ and hyperphosphorylated tau protein, significantly reducing their accumulation in the brain.^186^, ^187^ It is noteworthy that some Fe65 isoforms may indirectly influence amyloid plaque formation by modulating APP intracellular domain signalling. Fe65 also plays roles in neuronal growth,^188^ differentiation, synaptic function, cytoskeleton regulation,^189^ lipid metabolism and gene transcription.^190^, ^191^

#### 5.3.2 Integrated mitochondrial targeting, anti‐inflammatory and antioxidant therapy

**FIGURE 7:**
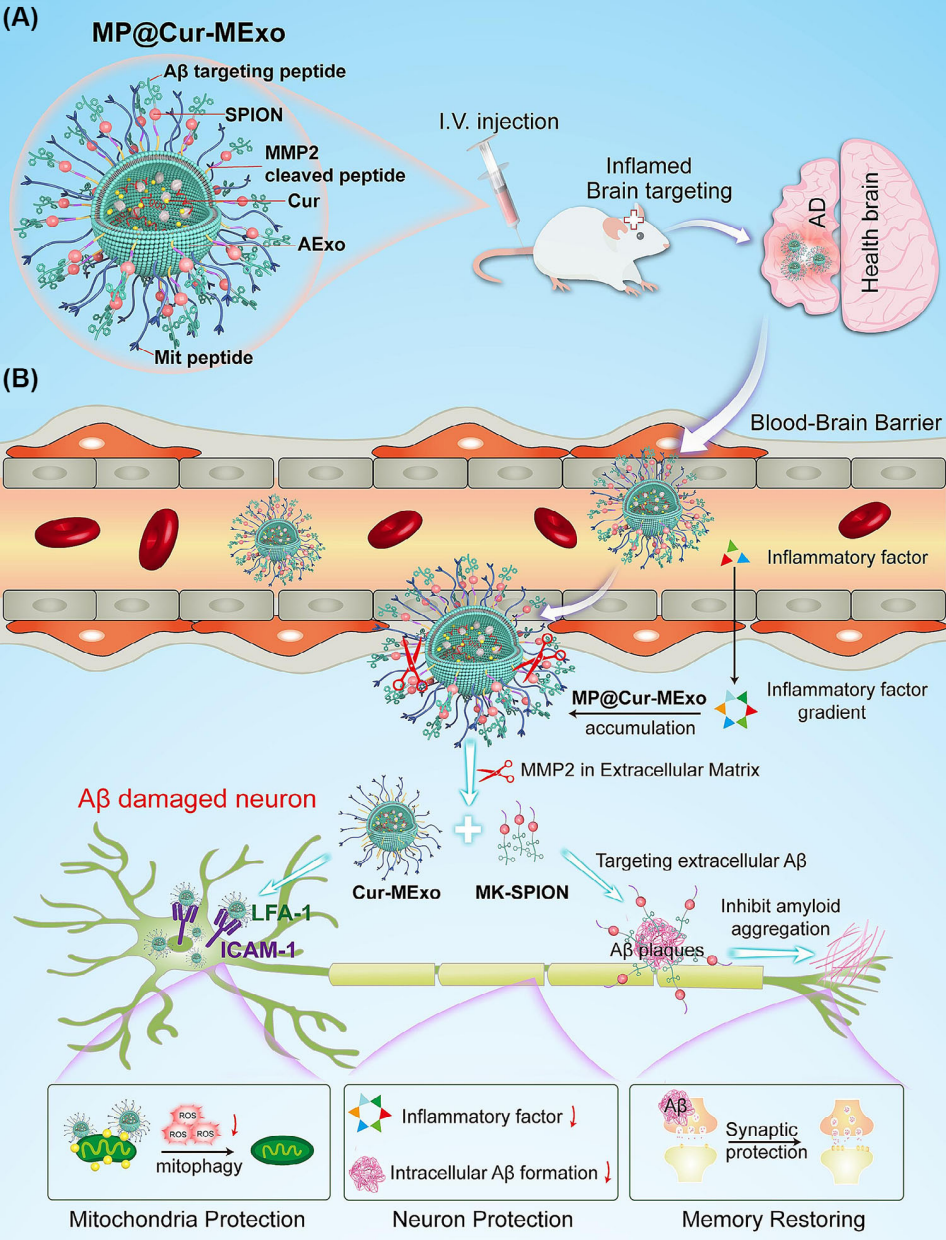
Schematic of multi‐targeted therapy for improving mitochondria health in amyloid‐beta (Aβ)‐activated neuron cells by inhibiting mitophagy. (A) The multi‐targeted engineered activated neutrophil exosomes (MP@Cur‐MExo) targeted the inflamed brain site of Alzheimer's disease (AD) mice after systemic administration in response to neuroinflammation. (B) MP@Cur‐MExo are exosomes derived from interleukin‐8 (IL‐8)‐stimulated neutrophils decorated with mitochondria targeting ligand and Aβ‐targeted superparamagnetic iron oxide nanoparticle (SPION). Engineered exosomes can selectively response to matrix metalloproteinase‐2 (MMP‐2) and release SPION and curcumin‐loaded AExo (Cur‐MExo), which can protect neuron cells against Aβ‐induced mitochondrial deficiency of energy metabolism, prevent intracellular Aβ production, inhibiting extracellular Aβ aggregation.

Figure 7 depicts the multifunctional engineered exosome (MP@Cur‐MExo) system, illustrating its components and therapeutic mechanisms in AD treatment. SPIONs, known for their utility in biomedicine,^192^ were modified with the Aβ‐targeting KLVFF peptide (which inhibit Aβ aggregation and reduce neurotoxicity)^193^ and a matrix metalloproteinase‐2 (MMP‐2)‐sensitive peptide (responsive to MMP‐2 overexpressed in the AD brain) to form MK‐SPIONs. A mitochondrial targeting peptide (MitP) was incorporated to guide exosomes to neuronal mitochondria and improve mitochondrial metabolic function.^194^ Curcumin (Cur), chosen for its lipophilicity, antioxidant and anti‐inflammatory properties, was encapsulated into exosomes via passive loading.^195^, ^196^

The construction process involved several steps. The KLVFF peptide (NHS‐PEG500‐KLVFF) and MMP‐2‐sensitive peptide (N3‐MMP2‐NHS) were conjugated to SPIONs to form MK‐SPIONs. Meanwhile, neutrophil‐derived exosomes, activated by IL‐8, were functionalised with DBCO groups by reacting DBCO‐sulpho‐NHS with amine groups on the exosome surface, yielding DBCO‐AExo. The DBCO‐modified exosomes were then conjugated to N3‐modified MK‐SPIONs and MitP via a click chemistry reaction, forming stable covalent bonds and anchoring these components to the exosomal surface.^80^ Finally, Cur was loaded into the exosomes by passive diffusion, resulting in the complete MP@Cur‐MExo system. Experimental results demonstrated that this engineered exosomes system significantly enhanced mitochondrial function, effectively inhibited Aβ aggregation, and reduced neuroinflammation in an AD mouse model by simultaneously engaging multiple key targets. These synergistic effects culminated in significant improvement of cognitive function, achieving the therapeutic goal for AD.

Notably, beyond conventional antioxidants such as Cur, epigenetic regulators such as the histone deacetylase 3 (HDAC3)‐specific inhibitor RGFP966 have also demonstrated potent anti‐oxidative and anti‐inflammatory effects by activating the Nrf2 pathway and suppressing NLRP3 inflammasome activation, as evidenced in traumatic brain injury model.^197^ This suggests that RGFP966 could serve as a promising candidate for exosomal encapsulation, complementing curcumin in a multi‐target therapeutic strategy. In summary, the application of engineered exosomes in AD treatment encompasses a logical hierarchy of strategies, from ensuring targeted delivery and controlled release to executing multi‐faceted therapeutic actions. These strategies are not mutually exclusive but are highly complementary. The most advanced therapeutic platforms often integrate elements from across these categories—for instance, a system that is brain‐targeted, microenvironment‐responsive and carries multi‐functional cargo. The potential inclusion of epigenetic modulators such as RGFP966—targeting both oxidative stress and neuroinflammation via mechanisms like the Nrf2 pathway—exemplifies the next generation of multi‐mechanistic exosome engineering. This integrated engineering approach holds immense promise for developing effective treatments capable of addressing the intricate and multifactorial pathophysiology of AD.

## 6 PRECLINICAL ADVANCES AND CHALLENGES

### 6.1 Preclinical research on engineered exosomes for AD

Preclinical research on AD from 2011 to 2025 has demonstrated encouraging therapeutic potential for engineered exosomes, as summarised in Table 5. The number of preclinical studies involving modified exosomes has steadily increased, with MSCs and immune cells—such as dendritic cells and macrophages—emerging as the predominant cellular sources. Exosomes derived from these cells offer therapeutic benefits due to their abundant availability, immunomodulatory properties, neuroprotective effects and inherent homing capability.^171^, ^198^ To enhance therapeutic efficacy, researchers have employed diverse engineering strategies, including receptor targeting (e.g., RVG‐LAMP2b binding to acetylcholine receptors),^199^ ligand/peptide conjugation (e.g., mannose targeting microglia)^155^ and nucleic acid aptamer modification (e.g., Aβ40‐specific aptamer for amyloid plaques recognition).^200^ Advanced cargo‐loading techniques such as electroporation, chemical coupling and membrane fusion have enabled the precise encapsulation of siRNA/miRNA (e.g., BACE1 siRNA^199^ and miR‐29b^201^) and small‐molecule drugs (e.g., curcumin^202^ and berberine^203^). These engineered exosomes have achieved significant therapeutic outcomes in preclinical AD models, including Aβ plaque clearance (via enhanced microglia phagocytosis or lysosome activation), suppression of neuroinflammation, restoration of synaptic plasticity and improvement of cognitive function.

**TABLE 5:** Preclinical applications of engineered exosomes in Alzheimer's disease (AD).

Year	Exosome source	Disease model	Targeting modification	Cargo	Loading method	Engineered product	Delivery route	Dose	Treatment regimen	Key findings	Reference
2011	Mouse bone marrow‐derived dendritic cells	Wild‐type C57BL/6 mice	RVG‐LAMP2b fusion	GAPDH siRNA, BACE1 siRNA	Electroporation	RVG‐LAMP2b‐modified exosomes	Intravenous (tail vein)	150 µg siRNA + 150 µg exosomes	Single administration	60% mRNA and 62% protein knockdown of BACE1; no significant immune response	^199^
2019	MSCs	APP/PS1 mice	RVG peptide conjugation	None	DOPE‐NHS conjugation	RVG peptide‐conjugated MSC exosomes	Intravenous (tail vein)	5 × 10^11^ particles	Monthly for 4 months	Improved cognition, reduced Aβ levels, attenuated neuroinflammation	^170^
2020	BMSCs and HEK‐293T cells	Aβ1‐42‐induced AD mice	miR‐29b precursor transfection	miR‐29b	Transfection	miR‐29b‐enriched exosomes	Intrahippocampal injection	10 µg	Single administration	Prevented Aβ‐induced spatial learning and memory deficits	^201^
2022	RAW264.7 macrophages	Aβ1‐42‐induced AD mice	DSPE‐PEG‐mannose conjugation	Gemfibrozil	Physical combination	Mannose‐modified exosomes (MExo‐Gem)	Intravenous (tail vein)	100 µg/ml	Daily for 7 days	Enhanced Aβ clearance, targeted microglia, increased lysosomal activity	^155^
2022	BMSCs	Aβ1‐42‐induced AD mice	Lentiviral SHP2 transfection	SHP2 protein	Transfection	SHP2‐overexpressing MSC exosomes	Intravenous (tail vein)	100 µg	Every 2 days for 2 weeks	Promoted mitophagy, improved BBB penetration, alleviated pathology and cognitive deficits	^204^
2023	HT22 neuronal cells	5xFAD mice	Fe65 protein modification	Cory‐B	Sonication	Fe65‐modified exosomes (Fe65‐Exo)	Intravenous (tail vein)	20 mg/kg	Every 2 days for 45 days	Induced autophagy, improved cognition and pathology	^185^
2023	Mouse bone marrow MSCs	APP/PS1 mice	AM1241 conjugation	None	Passive diffusion	AM1241‐loaded EVs (EVs‐AM1241)	Intravenous (tail vein)	6 mg/kg AM1241	Every 2 days for 2 weeks	Reversed neurodegenerative pathology and enhanced neurogenesis	^205^
2024	HEK293T cells	5xFAD mice	Light‐inducible mMaple3‐CD9 fusion	dCas9‐DNMT3A	Light induction	MAPLEX system	Intranasal administration	50 µg	Every 2 days for 27 days	Reduced Bace1 expression, lowered Aβ levels, improved cognition	^174^
2024	IL‐8‐stimulated neutrophils	APP/PS1 mice	LFA‐1/Aβ ligand + MMP‐2‐sensitive peptide	Curcumin, SPIONs	Chemical conjugation	MP@Cur‐MExo	Intravenous (tail vein)	1 × 10^8^ particles	Twice weekly for 2 weeks	Improved mitochondrial function, reduced Aβ‐induced damage, dual diagnostic/therapeutic potential	^80^
2024	Dendritic cells	3xTg‐AD mice	CD47/SST modification	miR‐29b‐2	Transfection	CD47/SST‐modified exosomes	Intravenous (tail vein)	2.5 × 10^10^ particles	Single administration	Targeted hippocampal SST receptors, reduced PSEN1 and Aβ1‐42 oligomer expression	^171^
2024	MSCs	Aβ1‐42‐induced AD mice	miR‐206‐3p antagomir loading	miR‐206‐3p antagomir	Electroporation	Antagomir‐loaded MSC exosomes	Intranasal administration	5.22 × 10^8^ particles	Every 2 days for 6 weeks	Enhanced neurogenesis, reduced Aβ deposition, improved synaptic plasticity	^172^
2025	SH‐SY5Y cells	AD microfluidic triculture system	None	miR‐124‐3p	Exo‐Fect transfection	Exo‐Fect‐engineered exosomes	Microfluidic device delivery	2.20 ± 1.54 × 10^7^ exosomes/µg	Single administration	Reduced neuronal apoptosis, remodeled microglia phenotype, alleviated astrocyte reactivity	^206^
2025	Plasma‐derived exosomes	TauP301S transgenic mice	C3 peptide and TPP conjugation	Curcumin	Liposome extruder, post‐insertion method	C3/TPP‐modified exosomes (C3/TPP‐Exo)	Intravenous (tail vein)	5 mg/kg	Single administration	Improved mitochondrial protection in neurons	^202^
2025	hUC‐MSCs	5xFAD mice	RVG29 peptide conjugation	BACE1 siRNA, berberine	Ultrasound‐assisted loading	RVG29‐conjugated exosomes	Intranasal administration	1 mg/mL	Twice weekly for 4 weeks	Improved cognitive function, promoted nerve repair, reduced inflammation, suppressed glial responses	^173^
2025	Microglia‐derived exosomes + liposomes	Aβ‐injected mice	Transferrin conjugation	Berberine, palmatine	Film dispersion, water bath	Transferrin‐modified hybrid EVs	Intravenous (tail vein)	Ber: 1.27 mg/kg; Pal: 1.60 mg/kg	Every 2 days for 16 days	Alleviated Aβ deposition, inflammation, and nerve damage; improved behavioural and cognitive functions	^203^
2025	Hybrid exosomes (BMVEC + macrophage)	APP/PS1 mice	Aβ40 nucleic acid aptamer (chol‐Apt40)	Polydopamine nanoparticles, resveratrol, Aβ‐targeted nucleic acid aptamers	Membrane fusion	RPDA@Rb‐A hybrid exosomes	Intravenous (tail vein)	Not specified	Every 3 days for 6 weeks	Degraded Aβ aggregates, promoted Aβ clearance, regulated microglia dysfunction	^200^

Abbreviations: Aβ, amyloid‐beta; APP, amyloid precursor protein; BACE1, beta‐secretase 1; BBB, blood‒brain barrier; BMSCs, bone marrow mesenchymal stem cells; BMVEC, brain microvascular endothelial cells; DOPE, 1,2‐dioleoyl‐sn‐glycero‐3‐phosphoethanolamine; EVs, extracellular vesicles; hUC‐MSCs, human umbilical cord‐derived mesenchymal stem cells; LAMP, lysosome‐associated membrane protein; MSCs, mesenchymal stem cells; MMP‐2, matrix metalloproteinase‐2; NHS, N‐hydroxysuccinimide; PEG, polyethylene glycol; PSEN1, presenilin 1; RVG, rabies virus glycoprotein; SPIONs, superparamagnetic iron oxide nanoparticles; SST, somatostatin; TPP, triphenylphosphonium.

The route of administration is critically important for CNS‐targeted therapy. Intravenous injection remains the most commonly used method (employed in 10 out of 16 studies). However, intranasal delivery is gaining increasing attention due to its non‐invasiveness and capacity to provide direct access to the CNS. A landmark clinical trial conducted by Wang Gang's team—the world's first exosome nasal spray trial for AD (NCT04388982)—has successfully demonstrated the safety and preliminary efficacy of intranasal exosome administration in AD patients, thereby providing strong support for subsequent clinical translational.^207^ Nevertheless, despite these promising preclinical results, several challenges remain for the clinical application of modified exosomes.

### 6.2 Clinical translation and feasibility of exosome‐based therapies

The robust preclinical foundation has paved the way for initial clinical applications, as reflected in the ongoing and completed trials detailed in Table 6. These studies represent a critical step in translating engineered exosomes from bench to bedside, particularly for AD and other neurological diseases.

**TABLE 6:** Summary of registered clinical trials investigating exosome‐based therapies for nervous system diseases.

NCT Number	Exosome source	Dose	Delivery route	Dosing frequency	Target disease	Study period (start–completion)	Phase	Status	Age inclusion	Enrollment	Follow‐up period
NCT03384433	Allogenic MSC‐derived miR‐124 enriched	Not provided	Stereotactic injection	NR	Acute ischaemic stroke	2019‐04‐17–2021‐12‐17	Phase 1/2	Completed	40–80 years	5	12 months
NCT04202770	Allogenic amniotic fluid derived	15 mL (unconcentrated)	Intravenous infusion	NR	Refractory depression, anxiety	2019‐12‐01–2024‐12 (Est.)	N/A	Suspended^a^	≥18 years	300	8 weeks
NCT04313647	MSC derived	2.0 × 10^8^ to 16.0 × 10^8^ nanoparticles/3 mL	Aerosol inhalation	Once daily	Healthy volunteers	2020‐03‐12–2020‐07‐31	Phase 1	Completed	18–45 years	24	7 days
NCT04388982	Adipose‐derived MSC exosomes	5–20 µg	Nasal drip	Twice weekly (12 weeks)	Alzheimer's disease	2020‐07‐01–2022‐08 (Est.)	Phase 1/2	Recruiting	≥50 years	9	48 weeks
NCT05886205	iPSC‐derived exosomes	2–18 µg	Nasal inhalation	Daily (12 weeks)	Focal epilepsy	2023‐06‐05–2025‐11‐13 (Est.)	Early phase 1	Recruiting	18–70 years	34	24 weeks
NCT06138210	Human iPSC‐derived exosomes	2 × 10^9^ to 8 × 10^9^ particles/kg	Intravenous infusion	Once daily for 7 days	Acute ischaemic stroke	2024‐05‐27–2025‐08‐30 (Est.)	Phase 1	Recruiting	18–70 years	29	90 days
NCT06598202	Umbilical cord blood MSC derived	1 mL	Nasal drop inhalation	Twice weekly (2 weeks)	Amyotrophic lateral sclerosis	2024‐12‐01–2026‐05‐30 (Est.)	Phase 1/2	Recruiting	18–80 years	38	24 weeks
NCT06612710	Neural stem cell‐derived EVs	4 × 10^9^ particles/kg	Intravenous injection	Once daily (7 days)	Ischaemic stroke	2024‐11‐30–2025‐11‐30 (Est.)	Phase 1	Not yet recruiting	18–75 years	69	24 weeks
NCT06607900	Umbilical cord MSC derived	Not provided	Nasal inhalation	NR	Multiple neurodegenerative diseases	2024‐11‐01–2028‐08‐31 (Est.)	Phase 1	Not yet recruiting	40–80 years	100	12 months

*Note*: All estimated completion dates are marked with ‘(Est.)’.

Abbreviations: EVs, extracellular vesicles; iPSC, induced pluripotent stem cell; MSC, mesenchymal stem cell; N/A, not applicable; NCT, national clinical trial; NR, not reported in the registry.

^a^ Suspended (pending product development status).

*Source*: ClinicalTrials.gov.

Clinical trials reflect a strategic selection of exosome sources that align with scalable and clinically feasible production systems. Allogenic MSCs (e.g., from adipose tissue or umbilical cord) are the most frequently used source, valued for their established safety profile and regenerative properties. Notably, the AD trial NCT04388982 utilises adipose‐derived MSC exosomes. Moreover, the emergence of iPSC‐derived exosomes (e.g., in trails NCT05886205 and NCT06138210) represents a significant advancement, offering a potentially unlimited and standardised supply of exosomes with consistent quality—a crucial factor for large‐scale clinical application.

Clinical trials are now validating administration routes previously highlighted in preclinical research. Intravenous infusion remains a standard approach for systemic delivery (e.g., NCT04202770, NCT06138210, NCT06612710). At the same time, the intranasal route is being actively investigated in multiple recruiting trials (NCT04388982, NCT05886205, NCT06598202, NCT06607900). This direct clinical evaluation of intranasal delivery—inspired by its preclinical advantages—confirms its feasibility in patients and its potential to achieve high CNS biodistribution with minimal systemic exposure.

Although current early‐phase clinical trials primarily focus on establishing safety and tolerability of naive or minimally modified exosomes, their success is a necessary precursor to introducing more complex engineered exosomes variants. The demonstrated feasibility of manufacturing, dosing and administering exosomes from various sources via different routes helps build the regulatory and practical framework for future therapies. This foundation will facilitate the subsequent integration of advanced engineering strategies—such as targeted ligands and therapeutic cargoes loading—that have shown high efficacy in preclinical models, as exemplified in Table 5.

Collectively, these early clinical experiences validate key preclinical insights while outlining a clear pathway forward. The following section will detail a systematic clinical translation roadmap designed to address the remaining challenges in standardisation, safety evaluation and trial design that must be overcome to fully realise the potential of engineered exosomes in AD treatment.

## 7 CLINICAL TRANSLATION ROADMAP

The promising preclinical outcomes of engineered exosomes necessitate a clear translational roadmap to bridge the gap between laboratory research and clinical application in AD. This section outlines the critical pathway for their clinical adoption.

### 7.1 Towards standardised and scalable GMP production

Scaling up exosome production from laboratory to clinical grade presents a formidable challenge, particularly in ensuring consistent quality across batches. The inherent heterogeneity of exosomes, which reflects the physiological status of parent cells—such as MSCs or dendritic cells—necessitates the implementation of standardised donor cell screening and rigorous quality control protocols.^208^, ^209^ A comprehensive quality monitoring system must be established throughout the entire manufacturing process, encompassing phenotypic stability of donor cells, secretome profiles and the functional activity of the final exosome product. To address these issues, induced pluripotent stem cell‐derived mesenchymal‐like cells (iPSC‐MSCs) have emerged as a promising alternative.^210^ By combining the unlimited proliferative potential of iPSCs with the therapeutic properties of MSCs, iPSC‐MSCs enable a three‐ to fivefold increase in exosome yield and exhibit more consistent biological characteristics compared to conventional cell sources.^211^ The establishment of standardised, GMP‐compliant cell banks represents a critical advancement, providing a traceable and uniform starting material that enhances the predictability of clinical outcomes.^212^

Implementation of this pillar necessitates a systematic, multi‐pronged strategy. First, a multidimensional evaluation framework for ‘seed cells’ should be established, integrating critical metrics such as genomic stability, epigenetic features and secretome profiling.^213^ Second, critical quality attributes of therapeutic exosomes must be clearly delineated through robust preclinical studies. These generally include particle concentration, size distribution, zeta potential, specific identity markers (e.g., CD63, CD81), purity (ensuring absence of protein aggregates or cellular debris), cargo loading efficiency and functional potency.^214^ Third, standardised operating procedures must be developed and strictly implemented across all stages—from cell culture and exosome isolation to purification and final formulation. Innovative platforms such as integrated microfluidic devices hold considerable potential for improving the precision and reproducibility of exosome engineering.^215^

Furthermore, the scalability and commercial translation of engineered exosomes face substantial intellectual property (IP) challenges. Core technologies—including CRISPR/Cas9‐mediated cargo loading and bioorthogonal chemistry for surface modification—are frequently constrained by complex patent landscapes and ongoing legal disputes. Such IP barriers may hinder clinical adoption and underscore the need for comprehensive freedom‐to‐operate analyses as well as potentially expensive licensing agreements. Consequently, proactive IP management—through approaches such as patent pooling, cross‐licensing and public‒private partnerships—is essential to de‐risk the translational pathway and prevent proprietary technologies from impeding the development of accessible AD therapies.^216^, ^217^

### 7.2 Comprehensive assessment of safety and immunogenicity

A comprehensive understanding of the safety profile is essential for any clinical candidate intended for the treatment of AD. Engineered exosomes entail distinct safety considerations that necessitate evaluation through a systematic and standardised framework. Systemically administered exosomes are susceptible to non‐specific uptake by the mononuclear phagocyte system, particularly in the liver and spleen, thereby raising potential concerns regarding acute and chronic organ toxicity.^218^ Although intranasal delivery reduces systemic exposure and associated risks, its long‐term safety profile—especially under conditions of chronic administration, which is likely required for AD—remains insufficiently characterised and warrants further investigation. Moreover, as natural carriers of bioactive molecules, exosomes must undergo rigorous purification to ensure the absence of endogenous neurotoxic proteins, such as Aβ and hyperphosphorylated tau, to prevent potential exacerbation of neuropathology.^219^, ^220^

Engineered modifications, particularly the surface display of heterologous proteins such as RVG‐LAMP2b for brain targeting, may elicit unintended immune responses, including the formation of anti‐drug antibodies (ADAs). For example, exosomes derived from commonly used cell lines such as HEK293T may carry residual host cell antigens, which could lead to therapeutic neutralisation or anaphylactic reactions upon repeated administration.^221^ Although the immunogenic risk is considerably lower compared to viral vectors, exosomes can effectively deliver encapsulated nucleic acids—such as siRNA targeting BACE1 or CRISPR‐based gene‐editing components—to recipient cells. The potential for off‐target effects, particularly in the case of exosomes carrying gene‐editing payloads, warrants rigorous evaluation in clinically relevant AD models.^222^

Therefore, a tiered safety assessment strategy specifically tailored to AD is recommended: (i) in vitro evaluations, including assays for immunocyte activation—such as measurement of cytokine release from peripheral blood mononuclear cells—and haemolysis testing.^223^ (ii) Preclinical in vivo toxicology studies involving both acute and chronic toxicity assessments in rodent models and, critically, in non‐human primates, with emphasis on histopathological analysis of primary clearance organs and the CNS. Evidence from studies in transgenic AD models, such as 5xFAD mice, has demonstrated improved cognitive function following intravenous administration of certain exosome formulations without significant toxicity, supporting their therapeutic potential.^185^ (iii) Long‐term monitoring: given the chronic nature of AD, clinical trial protocols should incorporate structured long‐term follow‐up plans to detect potential delayed adverse events. There is a critical need to cite and evaluate available immunogenicity data for targeted exosomes—particularly those bearing targeting ligands such as RVG—including reports of ADA detection in animal models. Additionally, reference to published or ongoing large‐scale, repeat‐dose toxicity studies of exosome‐based therapeutics would further strengthen the overall safety profile evaluation.^224^, ^225^

### 7.3 Informing future trials: insights and validation strategies

The clinical translation landscape for exosome‐based therapies for AD is beginning to emerge, offering critical insights and establishing important precedents for future clinical trials. The world's first clinical trial of an exosome‐based nasal spray for AD, conducted by Gang's team, has pioneered the clinical application pathway by successfully demonstrating the preliminary safety and feasibility of intranasal administration in patients with AD. A systematic search of clinical trial registries, such as ClinicalTrials.gov, reveals a growing yet still limited number of registered studies investigating exosome‐based interventions for AD. These trials predominantly involve exosomes derived from MSCs or iPSCs, administered via intravenous or intranasal routes, with primary endpoints focused on safety and tolerability, and secondary endpoints evaluating cognitive function and biomarker changes.

Success in future pivotal trials will depend on sophisticated trial designs that account for the complex and protracted nature of AD. There is a critical need to correlate treatment response with robust and accessible biomarkers. The analysis of blood‐based exosomal cargo, including synaptic proteins such as neurogranin and GAP‐43, Aβ and phosphorylated tau, holds substantial promise as both a pharmacodynamic and prognostic biomarker.^226^ Neurofilament light chain is increasingly recognised as a sensitive indicator of neuroaxonal injury in clinical trials for neurodegenerative disorders.^227^ Optimisation of administration regimens represents another key challenge; for routes such as intranasal delivery, determining the optimal dosing frequency, volume, and formulation to ensure consistent and sufficient CNS delivery is paramount.

Future trials should employ rigorous designs, including stringent enrollment criteria—such as enrichment for patients with specific biomarker profiles, including elevated Aβ or phosphorylated tau—along with extended long‐term follow‐up periods, and the use of multi‐modal efficacy assessments that integrate novel fluid biomarkers with advanced neuroimaging techniques (e.g., amyloid‐PET, tau‐PET) and sensitive cognitive test batteries.^228^ Drawing insights from recently successful AD therapeutic trials, which have heavily relied on biomarker‐enriched populations and imaging‐based surrogates, will be critical to demonstrating the clinical utility of engineered exosomes.^229^

## 8 CONCLUSIONS

Engineered exosomes offer a transformative and highly promising platform that enables an integrated, multi‐targeted strategy to address the complex and multifactorial pathology of AD. Through rational design, exosomes can be engineered with precise brain‐targeting capabilities and simultaneously loaded with diverse therapeutic molecules to synergistically modulate core pathological processes, including Aβ and tau aggregation, chronic neuroinflammation, and impaired neurotrophic support. This combinatorial approach not only overcomes the limitations of conventional single‐target therapies but also offers renewed potential for fundamentally altering disease progression.

However, the clinical translation and application of this technology still face significant challenges. Future work should prioritise the following directions: first of all, establishing robust, scalable and standardised production processes to ensure batch‐to‐batch consistency and high product quality; second, further refining loading and surface modification techniques to improve targeting accuracy and delivery efficiency; and third, most critically, implementing systematic evaluation of long‐term safety, immunogenicity and potential off‐target effects through rigorous preclinical and clinical studies.

With the emergence of innovative approaches such as hybrid‐cell exosomes and biomimetic synthetic vesicles, together with a deepening understanding of exosome biology, engineered exosomes will play a pivotal role in the future therapeutic landscape of AD, bringing renewed hope to millions of patients worldwide.

## AUTHOR CONTRIBUTIONS

*Writing—review and editing and methodology*: Chunlin Zou and Zhenhua Qiu. *Writing—review and editing, writing—original draft, methodology, investigation, formal analysis and data curation*: Yuehan Zhang. *Figure design and production, and formal analysis*: Zhenyang Li and Haien Guan.

## CONFLICT OF INTEREST STATEMENT

The authors declare they have no conflicts of interest.

## ETHICS STATEMENT

Not applicable.

## Data Availability

Data sharing is not applicable to this article as no new data were created or analysed in this study.
